# Mediterranean grassland soil C–N compound turnover is dependent on rainfall and depth, and is mediated by genomically divergent microorganisms

**DOI:** 10.1038/s41564-019-0449-y

**Published:** 2019-05-20

**Authors:** Spencer Diamond, Peter F. Andeer, Zhou Li, Alexander Crits-Christoph, David Burstein, Karthik Anantharaman, Katherine R. Lane, Brian C. Thomas, Chongle Pan, Trent R. Northen, Jillian F. Banfield

**Affiliations:** 1 Department of Earth and Planetary Science, University of California, Berkeley, Berkeley, CA USA; 2 Environmental Genomics and Systems Biology Division, Lawrence Berkeley National Laboratory, Berkeley, CA USA; 3 Oak Ridge National Laboratory, Oak Ridge, TN USA; 4 Department of Plant and Microbial Biology, University of California, Berkeley, Berkeley, CA USA; 5 Joint Genome Institute, Lawrence Berkeley National Laboratory, Walnut Creek, CA USA; 6 Department of Environmental Science, Policy, and Management, University of California, Berkeley, Berkeley, CA USA; 7 Present Address: School of Molecular Cell Biology and Biotechnology, Tel Aviv University, Tel Aviv, Israel; 8 Present Address: Department of Bacteriology, University of Wisconsin, Madison, WI USA; 9 Present Address: School of Computer Science and Department of Microbiology and Plant Biology, University of Oklahoma, Norman, OK USA

**Keywords:** Microbial ecology, Microbiology, Soil microbiology, Metagenomics

## Abstract

Soil microbial activity drives the carbon and nitrogen cycles and is an important determinant of atmospheric trace gas turnover, yet most soils are dominated by microorganisms with unknown metabolic capacities. Even Acidobacteria, among the most abundant bacteria in soil, remain poorly characterized, and functions across groups such as Verrucomicrobia, Gemmatimonadetes, Chloroflexi and Rokubacteria are understudied. Here, we have resolved 60 metagenomic and 20 proteomic data sets from a Mediterranean grassland soil ecosystem and recovered 793 near-complete microbial genomes from 18 phyla, representing around one-third of all microorganisms detected. Importantly, this enabled extensive genomics-based metabolic predictions for these communities. Acidobacteria from multiple previously unstudied classes have genomes that encode large enzyme complements for complex carbohydrate degradation. Alternatively, most microorganisms encode carbohydrate esterases that strip readily accessible methyl and acetyl groups from polymers like pectin and xylan, forming methanol and acetate, the availability of which could explain the high prevalence of C_1_ metabolism and acetate utilization in genomes. Microorganism abundances among samples collected at three soil depths and under natural and amended rainfall regimes indicate statistically higher associations of inorganic nitrogen metabolism and carbon degradation in deep and shallow soils, respectively. This partitioning decreased in samples under extended spring rainfall, indicating that long-term climate alteration can affect both carbon and nitrogen cycling. Overall, by leveraging natural and experimental gradients with genome-resolved metabolic profiles, we link microorganisms lacking prior genomic characterization to specific roles in complex carbon, C_1_, nitrate and ammonia transformations, and constrain factors that impact their distributions in soil.

## Abstract (web summary)

Using metagenomics and proteomics on Mediterranean grassland soil samples resulted in the recovery of 793 metagenome-assembled genomes and provided insights into microbial activity in this environment, and how this changes with soil depth and exposure to extended rainfall.

## Main

Grassland ecosystems cover 26% of all land area, store 34% of global terrestrial carbon and comprise 80% of agriculturally productive land^1,2^. Grasslands thus have a significant impact on global soil carbon storage, trace gas emissions and economic productivity^1,2^. Identifying microorganism capacities for carbon and nitrogen turnover is critical, as microorganisms ultimately determine how grassland soils cycle carbon and nitrogen and emit or absorb trace gases^3,4^ (In the context of this manuscript microorganisms only refers to Bacteria and Archaea.)

One of the biggest challenges in studying the metabolism of soil microbial communities is that most of the microorganisms have only been detected using 16S rRNA surveys^5,6^. While studies have been undertaken to link amplified metabolic genes or 16S rRNA gene abundances with soil trace gas fluxes or environmental conditions^7–11^, the large number of soil-associated microorganisms not represented by genomes precludes meaningful predictions of relationships between microorganism types and their biogeochemical functions.

The metabolic capacities of soil-associated microorganisms can be investigated if genomes can be reconstructed from soil samples^12–14^. However, this is notoriously difficult, as most soils have extremely high microbial diversity^15^. So far, few soil data sets have been even partially genomically resolved^13,16^, but recently it was shown that broad genomic resolution and community metabolic functions could be deduced in metagenomic studies targeting permafrost^12^.

Here, we have applied deep metagenomic sequencing and metaproteomic analyses to sub-root zone samples from a grassland soil ecosystem from a Mediterranean climate. Mediterranean grassland soils are of particular interest as they have not been genomically characterized and undergo strong seasonal drying and re-wetting that uniquely structures their microbial communities^17,18^. A subset of the soils in this study are currently undergoing a rainfall extension climate change experiment^19^. Despite the presence of thousands of species at low abundance levels and strain heterogeneity, we successfully reconstructed non-redundant draft-quality genomes that account for the majority of microorganisms detected by abundance. Overall, our data reveal important carbon and nitrogen turnover functions in understudied microbial groups, show a stark metabolic and phylogenetic stratification across soil depths, and support climate change as a factor that can significantly alter the carbon and nitrogen turnover capacity of soil microbial communities.

## Results

### Soil sampling and assembly

We collected 60 soil samples at 10–20 cm (just below the root zone), 20–30 cm and 30–40 cm depths from a grassland meadow within the Angelo Coastal Range Reserve in Northern California (Supplementary Fig. 1). Three of the six sampling sites had been subjected to over 14 years of rainfall amendment to simulate a predicted climate change scenario for northern California^19^. In total, we generated 1.2 Tb of raw read data, which assembled into 67 Gbp of contiguous sequence. Of this, 47 Gbp (70.2%) of the assembled sequences were >1 kb in length. On average, 36.4% of reads mapped back assemblies, and for some samples this mapping was as high as 64.7% (Supplementary Table 1).

### A species richness census reveals extensive sampling of soil microbial diversity

Although our approach overall is genome-centric, many microorganisms were at too low abundance to be represented by draft genomes. Thus, we used ribosomal protein S3 (rpS3) to conduct a census of the microbial diversity found at the site and to quantify relative organism abundances^20^. Across our 60 metagenomic assemblies we identified 10,158 rpS3 sequences (169 ± 93 per sample), which were grouped into 3,325 non-redundant clusters (see Methods) that approximate species groups (SGs) (Supplementary Table 2, Supplementary Fig. 2 and Supplementary Data 1 and 2).

Using our rpS3 sequences as phylogenetic markers we initially classified all of the microorganisms detected at the phylum and class levels. We detected 26 distinct phylum-level lineages, and the topology of the rpS3 tree suggested that most phyla are represented by few class level groups with high degrees of genus and species heterogeneity. We also found that the abundances of closely related microorganisms could be highly variable, differing in abundance by a factor of 10 (Supplementary Fig. 3 and Supplementary Data 3).

**Fig. 1:**
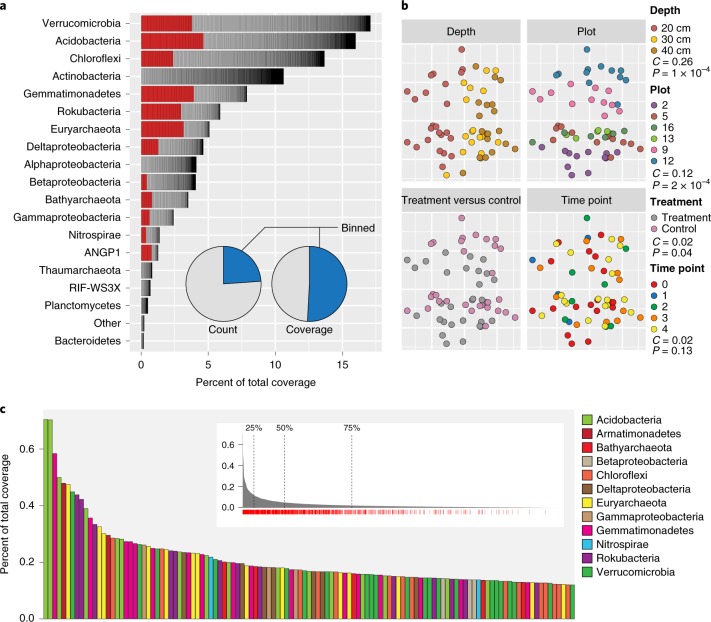
rpS3 species group abundance, influence of variables and abundance metrics. **a**, Percent of total coverage of all species groups (SGs) ranked by relative phylum coverage. ‘Other’ includes phyla with <5 SGs. Organisms in red are in the top 25% of organisms by coverage. Inset, pie charts showing the breakdown of SGs associated with genome bins (blue) based on count and coverage of SGs. **b**, NMDS plot (stress = 0.055) of SG UniFrac distances. The ordination is replicated and overlaid with the four data types collected across our 60 samples. Variable importance (*C*) and significance (*P*) calculated by an MRPP procedure are displayed in the key. **c**, Top 25% of SGs ranked by total coverage across all samples. Inset, full rank abundance curve showing the positions where 25%, 50% and 75% of the total data set coverage are reached. Red tick marks under the plot indicate SGs with bins. Also see Supplementary Table 5.

In agreement with many previous soil surveys^5,6^, we found that Verrucomicobia and Acidobacteria were the most relatively abundant lineages across our site (Fig. 1a). Generally, coverage was disproportionately concentrated in a small subset of SGs, and approximately 13% (443) of the detected microorganisms accounted for 50% of the total read coverage (Fig. 1c). Some microorganisms, such as specific Nitrospirae and Euryarchaeota, had high relative abundance despite their phylum as a whole exhibiting low relative abundance (Fig. 1a,c). Thus, while some phyla do not collectively account for a high fraction of the reconstructed microbiomes, individual microorganisms belonging to these phyla may be highly abundant.

### Spatial variation and treatment, but not time of sampling, contribute significant variance to microorganism abundance

To visualize the influence of depth, sampling location, sampling date and rainfall amendment on the abundance of SGs, we applied non-metric multidimensional scaling (NMDS) ordination to the weighted UniFrac distance matrix of SG coverage (Fig. 1b, Supplementary Tables 3 and 4 and Supplementary Data 4). Subsequently we used the multi-response permutation procedure (MRPP) to test the significance and strength of each variable’s influence. The results indicate that sampling depth, sampling location and rainfall amendment have significant effects on relative microorganism abundance and composition across samples (Fig. 1b). Sampling depth was the most influential factor (*C* = 0.26; *P* = 1 × 10^−4^), followed by sampling location (*C* = 0.12; *P* = 2 × 10^−4^) and rainfall amendment (*C* = 0.02; *P* = 0.04). While rainfall amendment showed a consistent effect, its effect occurs relative to sampling location (Fig. 1b). A large sample number was critical in observing this relationship, and was important for isolating the weaker effect caused by rainfall extension. However, we found that the date a sample was collected did not significantly influence overall SG variability, despite samples being collected over a 31-day period covering the transition from the dry to rainy season (Fig. 1b and Supplementary Fig. 1).

### A hybrid binning method resolved genomes from previously unsequenced lineages

Genomes reconstructed from each sample were used to link metabolic functions to specific microorganisms (see Methods). We recovered 10,463 genomic bins with an average of 174 ± 87 binned genomes per sample. After clustering bins based on the SGs assigned to their rpS3 gene, and filtering for estimated completeness of >70% and contamination of <10%, we recovered 793 unique microbial genomes (Supplementary Table 5).

Our reconstructed genomes represent 24% of SGs by number, but these genomes represent more than half (53%) of the SGs by total coverage (Fig. 1a). A total of 204 genomes were from microorganisms in the lowest quartile of total abundance (Fig. 1c). Importantly, we recovered 115 high-quality genomes (>95% estimated completeness) across 15 of the 26 microbial phyla detected at the site (Supplementary Table 5).

**Fig. 2:**
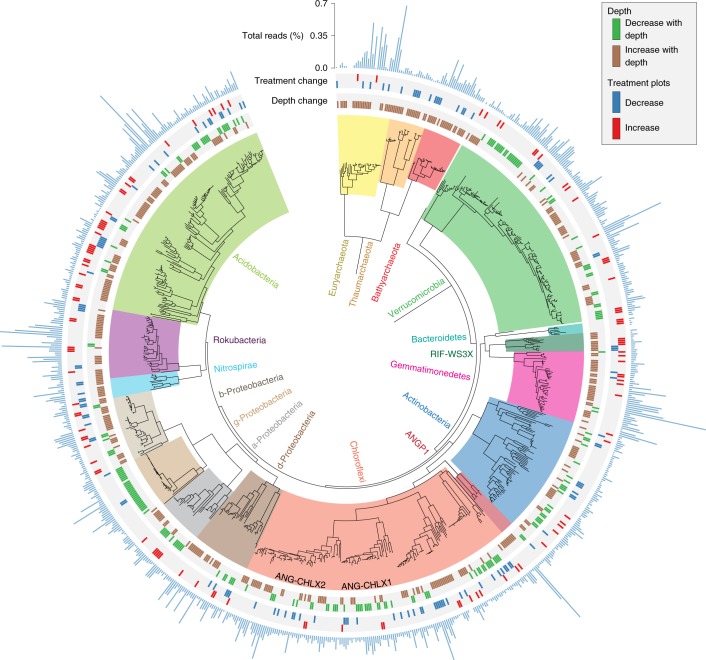
Maximum likelihood tree of all near-complete genomes. Phylogenetic tree constructed with a concatenated alignment of 15 co-located ribosomal proteins (L2, L3, L4, L5, L6, L14, L15, L16, L18, L22, L24, S3, S8, S17 and S19). The tree includes 722 bacterial and 71 archaeal genomes. The two Chloroflexi classes basal to classic Chloroflexi lineages are named. Concentric rings moving outward from the tree indicate if a genome’s associated SG abundance was found to significantly increase or decrease with depth and increase or decrease in plots under extended rainfall treatment at either 10–20 cm or 30–40 cm. For all genomes shown, the direction of response (increase or decrease) to extended rainfall treatment was never different between depths. The concentric bar plot indicates relative abundance (see Methods). For the complete ribosomal protein tree, see Supplementary Fig. 4 and Supplementary Data 5. For all exact relative abundance values and differential abundance statistics, see Supplementary Table 5.

A more detailed phylogenetic analysis using both a concatenated set of 15 ribosomal proteins (rp15) and 16S rRNA sequences indicated that we have significantly expanded the genomic coverage across a number of poorly sequenced soil lineages (Fig. 2, Supplementary Figs. 4 and 5, Supplementary Tables 5 and 6 and Supplementary Data 5–8). Many genomes from unsequenced lineages were relatively abundant microorganisms at our site. In particular, we recovered 145 near complete Acidobacterial genomes from 15 class-level lineages, four of which have no previously sequenced representative (Gp18, Gp5, Gp11 and Gp2) (Fig. 2 and Supplementary Figs. 4 and 5). We also found phylogenetic overlap between our Acidobacterial genomes and previously recovered but unclassified Acidobacterial genomes from a subsurface aquifer sediment in Rifle, Colorado^21^. By including genomes from both the Rifle and Angelo sites in our phylogenetic tree we were able to assign 17 genomes to Acidobacterial classes Gp7, Gp22 and Gp17, for which there was no previous class-level genomic information (Supplementary Fig. 5).

The majority of our Chloroflexi genomes came from four unsequenced or poorly sequenced class-level lineages. Nine genomes affiliate with a group referred to as CHLX from the Rifle aquifer sediment^21^, and 32 genomes phylogenetically place with a second lineage that includes one genome from Rifle sediment and one from arctic soil^13^. We also recovered 96 genomes from two class-level lineages within Chloroflexi with no previously sequenced representatives, hereafter referred to as ANG-CHLX1 and ANG-CHLX2 (Fig. 2 and Supplementary Fig. 4). The ANG-CHLX1 and ANG-CHLX2 clades form a strongly supported group basal to RIF-CHLX genomes and all known Chloroflexi lineages.

### The soil proteome indicates a high prevalence of C_1_, pentose sugar and small molecule metabolism

We used shotgun proteomic data from 20 samples to provide insight into abundant functions in situ (see Methods) and to guide or metabolic analysis of the reconstructed genomes. Overall, we identified 55,665 proteins with at least one uniquely mapped peptide that was detected with high mass accuracy. In total, 60% of the proteins identified could be assigned to one of 393 functional orthology groups (Supplementary Tables 7 and 8 and Supplementary Data 9).

The most abundant proteins identified were ABC transporters for sugars and amino acids, pentose sugar processing enzymes and enzymes degrading small C_1_ and nitrogen-containing compounds including formamidase, carbon monoxide dehydrogenase and methanol dehydrogenase (Supplementary Fig. 6 and Supplementary Results). A high abundance of xoxF-type methanol dehydrogenases had been previously reported from proteomics at this site^14^. In this study, we also detected high abundances of proteins annotated as carbon monoxide dehydrogenases (coxL), including coxL-TypeI, which functions in the oxidation of CO, and others. Genomic studies have indicated widespread distribution of diverse coxL subtypes in soils^9,22,23^, suggesting that subtypes other than TypeI may be important, and overlooked small molecule dehydrogenases with unknown specificity.

### Genome metabolic profiling identifies prevalent metabolism of small molecules and nitrogen cycling processes in unexpected microorganisms

Given the prevalence of enzymes that turn over low-molecular-weight compounds, we targeted their genes in our analyses of genome metabolic potential. The dbCAN and KEGG databases were used to profile reconstructed genomes^24,25^ (see Methods, Supplementary Figs. 7–9, Supplementary Tables 9–13 and Supplementary Data 10–14).

**Fig. 3:**
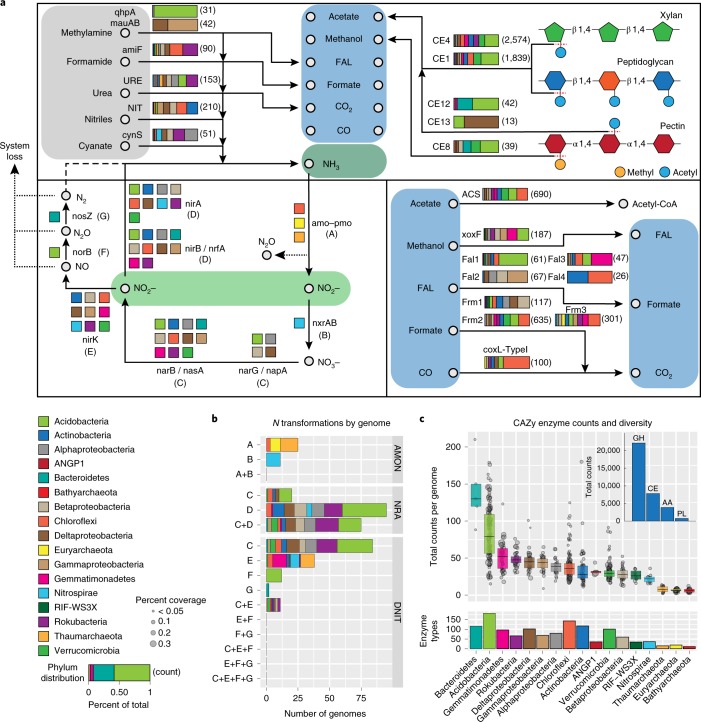
Predicted carbon and nitrogen metabolic transformations. **a**, Predicted phylum-level genomic capacity for breakdown of small carbon- and nitrogen-containing compounds, and liberation of methyl and acetyl groups from complex polymers. Horizontal bar plots indicate the fraction of genomes within a phylum encoding each function (as shown in the key on the bottom left). Numbers to the right of bars in parentheses indicate the total number of genes detected (*n* = 793 independent genomes). NIT, nitrilase; URE, urease; FAL, formaldehyde oxidation; ACL, acetyl-CoA synthetase. **b**, Counts of genomes encoding capacities for individual or multiple nitrogen transformation steps. AMON, ammonia oxidation to nitrate; NRA, nitrate reduction to ammonia; DNIT, denitrification (*n* = 793 independent genomes). **c**, Top, counts of carbohydrate active (CAZy) enzymes across genomes in each phylum. Points indicate the total counts in individual genomes and point sizes reflect genome relative coverage across all samples (as shown in the key on the bottom left). Box plots enclose 1st to 3rd quartiles of data values, with a black line at the median value. Top inset, bar plot showing the total number of CAZy enzymes across all genomes belonging to each CAZy class (GH, glycosyl hydrolase; CE, carbohydrate esterase; AA, auxiliary activity; PL, polysaccharide lyase). Bottom, count of all 246 possible CAZy enzymes types that were identified across a phylum (*n* = 793 independent genomes). Also see Supplementary Tables 10–13.

Methanol dehydrogenases were detected in 187 genomes, and all methanol dehydrogenases identified were of the XoxF type (Fig. 3a and Supplementary Fig. 7). These genes were abundant in Gemmatimonadetes and Rokubacteria, but also were detected in Gp1, Gp5 and Gp6 Acidobacterial genomes and four phyla of Proteobacteria (Fig. 3a). A total of 90 genomes encode formamidase (amiF), including 26 Chloroflexi and 30 Rokubacteria (Fig. 3a). Formamidase contributes to both formate and ammonia pools via the breakdown of formamide, which may originate from amino acid catabolism^25^. Using coxL as a marker for coxLMS-type CO dehydrogenases^9^ we detected 1,889 coxL homologues encoded in 466 genomes. However, only coxL-TypeI is known to metabolize CO^9,22^. We note that coxL-TypeI genes were encoded in 59 Chloroflexi genomes, with the majority being from ANG-CHLX1 and ANG-CHLX2 clades (Fig. 3a). However, the vast majority of coxL proteins were subtypes other than coxL-TypeI (Supplementary Fig. 8).

Bacteroidetes and Acidobacteria genomes encode the largest number of carbohydrate active enzymes (CAZy enzymes), but Acidobacteria far exceed Bacteroidetes in terms of both total genomes detected (152 versus 5) and relative abundance (16% versus 0.2% across all communities) (Figs. 2 and 3c and Supplementary Fig. 4). Acidobacteria also have the highest diversity of CAZy enzyme types, with 73% of CAZy families detected in at least one member of this phylum (Fig. 3c). Acidobacterial genomes from classes Gp1 and Gp3 are known to contain large numbers of CAZy enzyme genes^26^. Here we identified nine Acidobacterial classes containing genomes that encode >100 CAZy enzymes, including the previously unsequenced classes Gp2, Gp11 and Gp18 (Supplementary Fig. 10). This significantly expands the metabolic potential for complex carbohydrate turnover across the Acidobacteria phylum.

Across CAZy enzymes we noted a particularly high proportion of carbohydrate esterases (22%; Fig. 3a,c and Supplementary Fig. 10). Types CE1 and CE4 account for 56% of all carbohydrate esterases identified and liberate acetate from a broad spectrum of complex plant and microbial polymers^27,28^. Of the 793 genomes analysed, 81% contain either CE1 or CE4 as well as an encoded acetyl-coA synthetase to incorporate liberated acetate (Supplementary Fig. 10)

In analysing the genomic capacity to mediate inorganic nitrogen transformations we found most microorganisms only encode a single transformation reaction, and that nitrite is the most common reaction substrate (Fig. 3a,b and Supplementary Table 10). We did not detect any genome with the potential for complete denitrification, or complete nitrification via ammonia oxidation (Fig. 3b). Also, we found only two genomes classified as Bacteroidetes encoding the enzyme nosZ, which may indicate limited N_2_O turnover potential in this system (Fig. 3b).

Of the 49 genomes encoding nirK, 12 were Gemmatimonadetes, a genomically undersampled phylum that is not normally linked to nitrite conversion to nitric oxide (Fig. 3b). Many Gemmatimonadetes with nirK were also relatively abundant (Supplementary Table 10). The gene *norB*, which converts nitric oxide (NO) to N_2_O, was exclusively found within genomes of Acidobacteria (Fig. 3b). While five acidobacterial classes had previously been reported to encode *norB*^29^, we additionally detected these genes in Acidobacteria from Gp4, Gp5 and Gp13, suggesting a widespread capacity for nitric oxide reduction across the acidobacterial phylum (Supplementary Table 10).

### Microorganisms are phylogenetically and functionally stratified by depth

**Fig. 4:**
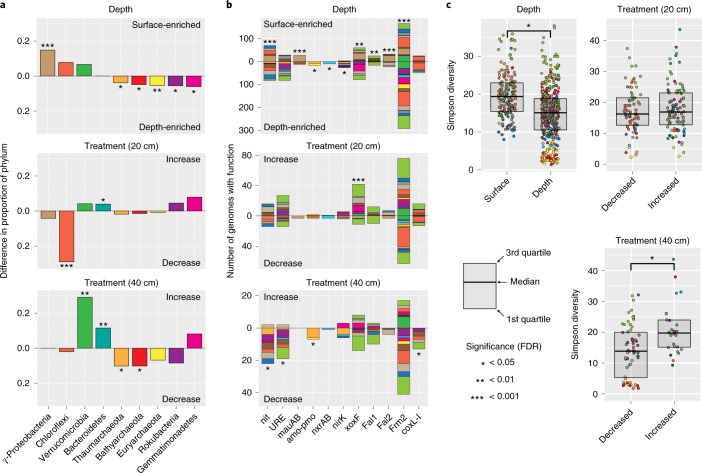
Enrichment of phyla and metabolic functions across depth and treatment. **a**, The difference in proportion of a phylum between genome groups that increase and decrease with depth/rainfall extension. Black asterisks indicate a significant enrichment of the phylum and bar direction indicates the genome set where the enrichment was found (two-sided permutation test: *false detection rate (FDR) ≤ 0.05, **FDR ≤ 0.01, ***FDR ≤ 0.001). **b**, Count of genomes encoding targeted carbon- and nitrogen-processing functions found to be significantly enriched in at least one comparison between genome groups that increase and decrease with depth/rainfall extension treatment. Genome counts only include those that were statistically different between depth or treatment shown. Black asterisks indicate a significant enrichment of the function and bar direction indicates the genome set where the enrichment was found (two-sided permutation test: *FDR ≤ 0.05, **FDR ≤ 0.01, ***FDR ≤ 0.001). Colours indicate phyla (see Fig. 3 for key). **c**, CAZy enzyme Simpson diversity distributions between genome groups that increase and decrease with depth/rainfall extension treatment. Simpson diversity has been transformed to the inverse form (1/(1 − Simpson)) for ease of viewing. Points are coloured by phylum (see Fig. 3 for key). A black asterisk between box plots indicates a statistical difference (two-sided Wilcoxon test: * FDR ≤ 0.05). Across all panels sample numbers were *n*_depth_ = 60 biologically independent samples, *n*_*2*0 cm treatment_ = 24 biologically independent samples and *n*_40 cm treatment_ = 20 biologically independent samples. Across all panels the numbers of genomes analysed were *n*_depth_ = 570 independent genomes, *n*_20 cm treatment_ = 173 independent genomes and *n*_40 cm treatment_ = 85 independent genomes. All tests were corrected for multiple testing using FDR. For all exact FDR values, see Supplementary Tables 14–16.

A total of 391 genomes significantly increased and 179 decreased in abundance with increasing soil depth. Thus, the majority of assembled genomes (72%) exhibit abundance patterns stratified by depth (Fig. 2 and Supplementary Table 5). All Archaeal lineages as well as Rokubacteria and Gemmatimonadetes were preferentially enriched in deeper samples, whereas Gammaproteobacteria were enriched at shallower depth (Fig. 4a and Supplementary Table 14).

Carbon and nitrogen turnover functions in the differentially abundant genome groups also exhibited stark depth-stratified patterns. C_1_ processing capacity and CAZy enzyme diversity were elevated in genomes more relatively abundant near the surface, while inorganic nitrogen turnover functions were enriched in genomes more relatively abundant in deeper soil (Fig. 4b,c and Supplementary Tables 15 and 16). We note that all Archaea had very low CAZy diversity, so we conducted a separate CAZy diversity analysis with Archaea removed. This additional analysis of only bacterial genomes indicates that genomes with higher relative abundance at depth still harbour a significantly reduced CAZy diversity compared to genomes more relatively abundant near the surface (Supplementary Fig. 11).

### Extended rainfall decreases soil depth-based functional stratification

Sample sets collected from 10–20 cm and 30–40 cm depths were analysed for rainfall extension effects separately to control for the strong phylogenetic and metabolic signal observed with depth. In response to rainfall extension, at 10–20 cm, 101 microorganisms increased and 72 microorganisms decreased in abundance, respectively (Supplementary Table 5). At 10–20 cm, the group of microorganisms increasing in abundance was enriched in Bacteroidetes whereas the group that decreased in abundance was enriched in Chloroflexi. At 30–40 cm, 26 microorganisms increased in abundance and 59 decreased. The group of microorganisms increasing in abundance at 30–40 cm was enriched in Bacteroidetes and Verrucomicrobia, whereas the group that decreased in abundance was enriched in Thaumarchaeota and Bathyarchaeota. Thus, in response to rainfall extension, we observe an enrichment of lineages associated with complex carbon degradation at both depths and a decrease of archaeal lineages in the 30–40 cm samples (Fig. 4a and Supplementary Table 14).

Metabolic profiling showed enrichment of methanol dehydrogenase in genomes of microorganisms that increased in abundance at 10–20 cm with extended rainfall (Fig. 4b and Supplementary Table 15). At 30–40 cm, there were statistically higher numbers of inorganic carbon- and nitrogen-processing functions including carbon monoxide dehydrogenase, nitrilase, urease and ammonia monooxygenase in genomes of microorganisms that decreased in abundance with treatment (Fig. 4b and Supplementary Table 15). However, at 30–40 cm, organisms increasing in abundance in response to treatment had genomes with a statistically higher CAZy enzyme diversity than those that decreased in abundance (Fig. 4c and Supplementary Table 16). However, the CAZy diversity analysis on only bacterial genomes found no significant difference, suggesting that the extended rainfall treatment does not specifically select for microorganisms with higher CAZy diversity, but instead selects against microorganisms with very low CAZy diversity (Supplementary Fig. 11). Thus, rainfall extension appears to increase C_1_ processing potential closer to the soil surface while causing a decrease in inorganic carbon- and nitrogen-processing potential at deeper depth. However, the decreased potential for processing inorganic carbon and nitrogen at depth is accompanied by a shift towards microbes with broader complex carbohydrate degradation potential.

## Discussion

We have recovered genomes for >50% of detected microorganisms in a grassland soil, based on coverage (which is a measure of cells sampled) (Fig. 1a), and significantly expand the availability of genomes for soil microorganisms from poorly sampled phyla. We provide evidence that metabolic systems for processing C_1_ compounds were relatively abundant and phylogenetically widespread, suggesting their importance in these soils (Fig. 3a). Additionally, we identified unexpected phyla encoding inorganic nitrogen turnover functions, and show that carbon and nitrogen metabolism is highly stratified across soil depths. It is also evident that climate alteration not only shifts community composition but alters the abundance of functions for important carbon and nitrogen biogeochemical cycling reactions.

Lanthanide-cofactor-bearing XoxF-type methanol dehydrogenases were highly prevalent, and the only methanol dehydrogenase class identified at our site. Thus, we conclude that lanthanides can be important mediators of carbon turnover in some soils. Lanthanides are often sequestered into phosphate minerals^30,31^ with low biological availability^32,33^, and their acquisition probably requires strong complexation of lanthanide ions by secondary metabolites such as siderophores^34^. In a recent report analysing a subset of genomes from this site, it was found that Gemmatimonadetes, Rokubacteria and Acidobacteria harbouring large numbers of XoxF sequences also exhibit extensive capacity for secondary metabolite biosynthesis^35^. Thus, we suspect a link between the prevalence of lanthanide-requiring enzymes and capacity to biosynthesize diverse secondary metabolites that promote mineral dissolution.

Finding credible type-I coxL CO dehydrogenases across many phyla supports CO as an important C_1_ energy source in soils^36^, and expands the microorganism range probably performing CO oxidation. However, many coxL-like sequences identified were phylogenetically unrelated to genuine type-I coxL sequences and probably have other substrates (Supplementary Fig. 8). Many molybdoprotein dehydrogenases act on small molecules like nicotinate and succinate^37,38^, which constitute large fractions of plant exudates^39^. Thus, we suggest that these enzymes may play roles in plant exudate processing and turnover in the studied soils. Future research may establish that these enzymes are currently under recognized mediators of small molecule turnover in soils.

Our data show that Gp2 Acidobacteria, which are abundant in some soils^40^, encode large repertoires of CAZy enzymes (Supplementary Fig. 10), and thus may represent an important and overlooked complex carbohydrate turnover sink. Additionally, the high prevalence of carbohydrate esterases we detected, as well as the genomic co-occurrence of acetate metabolism, suggests C_1_ compounds and small organic molecules are important and readily available carbon currencies for diverse microorganisms. As methyl and acetyl groups are common additions to many polymers^27,28^, the widespread prevalence of carbohydrate esterases may represent a strategy where readily available C_1_ and C_2_ carbon is accessed with minimal energetic investment. This observation may explain, in part, why low-molecular-weight carbon molecules are important currencies in this ecosystem.

The observation that most microorganisms encoding inorganic nitrogen turnover functions only harbour single steps of these pathways (Fig. 3b) parallels a similar finding for complex subsurface microbial communities^21^. Thus, both soils and sediments may be structured by metabolic handoffs, leading to high degrees of inter-organism cooperativity. Additionally, the identification of Gemmatimonadetes with the capacity for nitrite to nitric oxide reduction, and only two genomes with N_2_O processing capacity, shows denitrification in these soils differs from observations in other soil types^41,42^. These differences may directly impact the release of the climate-change relevant gases N_2_O and NO from this system.

We found that grassland soils can be highly stratified both phylogenetically and functionally. Additionally, deeper soils were significantly enriched in microorganism groups that are underrepresented in genomic databases. These findings have broad implications for understanding soil organic matter (SOM) turnover, as it is known that deeper strata account for a much larger fraction of SOM, with a much longer turnover time than SOM in shallow soil^43^. Thus, the genomes reported here contribute significantly to understanding the bacteria and archaea that could exert critical controls on the turnover rates of carbon stored in deeper soils.

The enrichment of enzymes involved in complex carbon metabolism, C_1_ and small molecule turnover in microorganisms closer to the surface (Fig. 4b,c) suggests that metabolic strategies at shallow depths are structured around plant-derived exudates and complex carbon. These data support the observation that SOM has a significantly shorter residence time closer to the soil surface^43^. In contrast, most inorganic nitrogen transformation functions are more prevalent or exclusively found in microorganisms enriched at greater depth (Fig. 4b). Thus, N_2_O discharged to the atmosphere from Mediterranean grasslands may originate from deeper soil strata.

Under the treatment involving extended spring rainfall, the relative decrease in microorganisms at deeper depths performing ammonia liberation and oxidation suggests a mechanism by which climate change could limit nitrogen cycling and N_2_O release. Simultaneously, increased complex carbohydrate degradation capacity at depth could counter this climate change impact by increasing CO_2_ release from previously recalcitrant SOM. However, the kinetics of CO_2_ and N_2_O release in response to rainfall changes, and the generality of these findings to other soils, remain uncertain. What is certain is that climate change can have a direct impact on the relative abundance and metabolic capacities of microorganisms in soil ecosystems, with potentially important impacts for trace gas release.

## Methods

### Study site and rainfall amendment

Soil samples were collected from three paired 70 m^2^ circular plots at the south meadow field site on the Angelo Coast Range Reserve in northern California (with permission given from APP# 27790; 39° 44′ 21.4′′ N 123° 37′ 51.0′′ W). One plot of each pair was part of an ongoing spring rainfall extension experiment initiated in the year 2000^19^, in its 14th year at the time of sampling. The rainfall extension experiment was established based on California rainfall patterns for the upcoming 50–100 years predicted by the Hadley Center for Climate Prediction and Research and the Canadian Center for Climate Modeling and Analysis in the year 2000. For plots that were treated with extended spring rainfall, every third day for three months during April–June, 14–16 mm of water was added over the ambient climate, reflecting a 20% increase in mean precipitation^19^. Amended water was collected from a mountain spring above the meadow; this was selected because its nitrogen and trace mineral concentrations fall within the range of concentrations for natural rainwater at the site.

Edaphic factors from the soil plots sampled have been reported in detail previously with respect to depth and spring rainfall extension treatment^44^. Briefly, the sampled soils are composed of roughly 50% sand, 30% silt and 20% clay and their pH ranged between 5.34 and 5.68. Carbon concentration and C:N ratio significantly decreased with depth and significantly increased under extended spring rainfall conditions. Carbon concentration was 18 mg g^−1^ (10 cm) to 6 mg g^−1^ (50 cm) under control conditions and 26 mg g^−1^ (10 cm) to 14 mg g^−1^ (50 cm) under extended rainfall. The C:N ratio was 11.2 (10 cm) to 8.1 (50 cm) under control conditions and 12.4 (10 cm) to 10.8 (50 cm) under extended rainfall. These measurements are in line with recently conducted pH and soluble organic carbon measurements taken from untreated soil at a depth of 10–40 cm at the north end of the meadow^14^.

### Sampling and DNA extraction

Samples were collected on five separate days beginning in September 2014 and ending in October 2014, before and following autumn rainfall events, as detailed in Supplementary Fig. 1 and Supplementary Table 1. Collection was undertaken across three biological replicate paired plots at the south end of the meadow. Each plot pair consisted of one biological control plot and one plot that was amended with extended spring rainfall, as detailed in ref. ^19^. Before starting a sample borehole, leaf litter and surface plant biomass were cleared from the sampling location. Subsequently, we used a manual soil coring device containing a sterilized 1.5 in × 7 in cylindrical polycarbonate insert to remove 10 cm of soil at a time from an individual sampling bore. Soil from the first 0–10 cm of each bore was discarded, and each subsequent 10 cm soil fraction was collected with a fresh sterilized insert. In the field after collection, the soil was immediately removed from the insert, homogenized, put into sterile bags and flash frozen on a mixture of dry ice and ethanol. In total, 60 soil samples were collected across all depths, plots and treatments. Soil samples were then maintained at −80 °C before DNA extraction.

For each sample, DNA was extracted from 10 g of homogenized soil using the PowerMax Soil DNA isolation kit (MoBio Laboratories) as described previously^14^. Metagenomic library preparation and DNA sequencing were performed at the Joint Genome Institute. Metagenomic libraries were prepared for sequencing on an Illumina HiSeq2500 platform, producing 250 bp paired-end reads with a target inter-read spacing of 500 bp. Raw sequencing data were subsequently processed with the Illumina CASAVA pipeline version 1.8.

### Metagenomic assembly

Raw reads were initially assessed for quality using the FastQC analysis suite (http://www.bioinformatics.babraham.ac.uk/projects/fastqc/). FastQC analysis indicated that for some samples a >1% GC bias existed in the last 50 bp of reads, so reads were all initially hard trimmed to a maximum of 200 bp using BBduk (https://sourceforge.net/projects/bbmap/) with the following parameters: forcetrimright = 200. Hard trimmed reads were then processed to remove Illumina adaptor sequences and phiX sequence contamination using BBduk with default parameters. Finally, reads were quality trimmed with Sickle using default parameters (https://github.com/najoshi/sickle).

The 60 samples were individually de novo assembled on a 24-core Intel Xenon Linux cluster node with 256 Gb of RAM using IDBA-UD v1.1.1^45^ with the following initial parameters: –pre_correction –mink 30 –maxk 200 –step 10. In the 12 cases where assemblies did not complete due to memory requirements, minimum k-mer size was increased to 40 bp and step size was increased to 20 bp. In the 14_0903_13_30cm sample where these parameters still did not allow the assembly to complete due to memory requirements, assembly was performed using megahit with the following parameters: –k-min 41 –k-max 201 –k-step 20 –min-contig-len 1000. The contigs resulting from the megahit assembly were then scaffolded using the IDBA-UD scaffolder with the following parameters: –seed_kmer 100 –min_contig 1000. Sequencing coverage of each contig was calculated by mapping raw reads back to assemblies using Bowtie2^46^ (see also Supplementary Table 1).

### Metagenome annotation

Following metagenome assembly, all samples were filtered to remove contigs smaller than 1 kb using pullseq (https://github.com/bcthomas/pullseq). Open reading frames (ORFs) were then predicted on all contigs using Prodigal v2.6.3^47^ with the following parameters: -m -p meta. Predicted ORFs were initially annotated using USEARCH^48^ to search all predicted ORFs against Uniprot^49^, Uniref90 and KEGG^25^. 16S ribosomal rRNA genes were predicted using the 16SfromHMM.py script from the ctbBio python package using default parameters (https://github.com/christophertbrown/bioscripts). Transfer RNAs were predicted using tRNAscan-SE^50^. The full metagenome samples and their annotations were then uploaded into our in-house analysis platform, ggKbase, where they are publically available (https://ggkbase.berkeley.edu).

### rpS3 identification, clustering and diversity analysis

rpS3 marker sequences were identified across all metagenomes using a custom hidden Markov model (HMM) based on an alignment of rpS3 sequences from the tree of life data set from ref. ^51^. Briefly, all rpS3 sequences provided in ref. ^51^ were initially filtered to remove Eukaryotic sequences. Sequences were then clustered at 90% ID using USEARCH with the following parameters: usearch -cluster_fast rpS3_sequences.faa -sort length -id 0.90 -maxrejects 0 -maxaccepts 0 -centroids rpS3_sequences_NR90.faa. The non-redundant sequences were then filtered to remove sequences <200 amino acids in length with pullseq. The resulting 2,249 sequences were aligned using muscle^52^ and an HMM was constructed from the alignment using HMMER3 with default parameters^53^. The HMM was benchmarked against the Uniprot reference proteomes database, and it was determined that rpS3 sequences could be confidently identified above a cutoff HMM alignment score of 40.

Across all metagenomes we identified a total of 10,159 rpS3 sequences that passed our HMM score threshold of 40. We clustered these sequences at 99% ID using USEARCH to obtain groups that roughly equate to species. We refer to these as species groups (SGs). The following USEARCH options were used: -cluster_fast all_rpS3.fa -sort length -id 0.99 -maxrejects 0 -maxaccepts 0 -centroids all_rpS3_centroids.faa. Subsequently we identified the longest contig in each rpS3 protein cluster to serve as a mapping target for abundance quantification of each SG (Supplementary Table 3 and Supplementary Data 2).

The longest contig representing each SG was mapped against the reads of each sample using Bowtie2 with default parameters. Mapped reads were filtered to remove all paired reads that mapped with <99% ID in either read pair. Reads mapped per contig were then counted to produce a read count table (Supplementary Table 3), and per base pair coverage was calculated to produce a coverage table (Supplementary Table 4). The coverage table was then normalized to the sequencing depth of each sample with the following formula: ((coverage / reads sequenced in sample) × 100,000,000) (Supplementary Table 5). For the purposes of quantifying the number of detected SGs per sample we considered an SG to be present if ≥2 reads were mapped to its longest contig at the 99% ID threshold.

To produce a collectors curve, we randomly selected from 1 to 60 samples without replacement using 100 sampling iterations at each sampling size. The number of unique SGs actually assembled (not just detected) in the sample subsets was quantified. We then fit a self-starting lomolino model^54^ to the data using the vegan package in R^55^. From this model fit we determined the slope of the collectors curve at 60 samples as well as extrapolated the total number of SGs and number of additional SGs per sample we would recover had we doubled our sampling efforts to 120 samples over the same sample set (Supplementary Fig. 3d,e). Using the unfiltered read count table as input we also calculated species richness estimators (Supplementary Fig. 3c), including the iChao2 metric^56^, with the SpadeR package in R (https://github.com/AnneChao/SpadeR/).

rpS3 SGs were classified at the phylum and class level (where possible) by constructing a phylogenetic tree containing our sequences and rpS3 reference sequences from ref. ^51^. Briefly, our 3,325 representative rpS3 sequences were concatenated with a set of 2,324 reference rpS3 sequences from ref. ^51^ and aligned using muscle^52^. The resulting alignment was stripped of columns containing >95% gap positions and a phylogenetic tree was constructed from the alignment using FastTree^57^. Sequences were then manually assigned phylum and class level lineage information based on their position relative to reference sequences in the tree.

### Ordination and variable importance analysis

All ordination and variable importance analysis was performed in R using the vegan and phyloseq packages^55,58^. SG coverage values were Hellinger standardized, and then SGs were removed that had a coefficient of variation (CV) of normalized coverage >3 or with <5 samples where raw coverage was ≥0.25. A maximum likelihood phylogenetic tree for weighted UniFrac (wUniFrac) was produced from a muscle alignment of all rpS3 SG centroids using IQ-TREE^59^ (Supplementary Data 4). The phylogenetic tree and normalized coverage table were then loaded into phyloseq where wUniFrac distance was calculated using the UniFrac command in phyloseq with the following parameters: weighted = TRUE, normalized = TRUE. NMDS ordinations were constructed from wUniFrac distance using the metaMDS command in vegan with the following options: k = 2, try = 500, trymax = 500 (NMDS stress = 0.055). Ordinations were plotted in R using ggplot^60^. The importance of metadata variables on community composition was calculated from wUniFrac distances using the mrpp command in vegan with the following options: permutations = 10000, weight.type = 1.

### Differential abundance analysis

Differential abundance of SGs across sampling depth and between treatment and control conditions was determined using raw read count data as the input (Supplementary Table 3) for the DEseq2 package in R^61^. We did not filter count data as DEseq filters low count data, and explicitly requests unfiltered data to more accurately estimate sample size factors and negative binomial model dispersion. To avoid linear combinations between DEseq model terms, paired plots from the same biological replicate were combined into a variable called ‘replicate’ (plot 2 & plot 5 = A; plot 9 & plot 12 = B; plot 13 & plot 16 = C).

Differential abundance of SGs across depth was tested by comparing a full DEseq model (design = ~Plot + Time_Point + Treat_Control + Depth) against a reduced DEseq model, where depth was omitted as a variable (reduced = ~Replicate + Time_Point + Treat_Control), using the likelihood ratio test (LRT). Resulting *P* values from the LRT were then corrected using the Benjamini and Hochberg procedure via false discovery rate (FDR) estimation^62^, and filtered to remove results with FDR > 0.05. We then fit individual linear models to the log normalized counts of each SG, showing a significant relationship with depth to determine an overall increase or decrease across the depth series. Models were fit using the lm function in R with the following form: log_count ~ depth. Model slopes and slope *P* values were subsequently extracted. Slope *P* values were corrected using FDR and values with FDR > 0.05 were removed. SGs with significant positive and negative model slopes were considered to increase and decrease with depth, respectively.

Differential abundance between treatment and control plots was analysed individually for each depth due to the extremely strong stratifying effect of depth. To contrast treatment and control at individual depths, a combined treatment–depth variable was created called ‘Factor’ (that is, treatment20cm versus control20cm). A DEseq object was constructed using the following form: ~Replicate + Time_Point + Factor. DEseq was then run using the standard negative binomial Wald test for GLM fits with the following options: fitType = ‘local’. Results where treatment and control conditions were contrasted for each depth were extracted and filtered to remove SGs with FDR > 0.05.

### Proteomics methods, annotation and analysis

A representative subset of 20 of our soil samples were selected for full proteome analysis, which was performed at the Oak Ridge National Laboratory. To be as representative as possible, samples were selected from the deepest and shallowest depths sampled from the two most geographically separated soil plots, from control and extended rainfall treated plots, and from sampling dates that occurred before and after rainfall events (Supplementary Fig. 1).

Proteins were extracted from each soil sample by using a previously described method^14^. Briefly, for each soil sample, the NoviPure Soil Protein Extraction Kit (MoBio Laboratories) was used to extract proteins from 10 g of soil. A crude protein extract was concentrated from 12 ml to 1 ml by using a 30 kDa Amicon Ultra-4 Centrifugal Filter Unit (Millipore). Proteins were then precipitated by trichloroacetic acid (Sigma-Aldrich) overnight at 4 °C and pelleted by centrifugation. Protein pellets were washed with ice-cold acetone (Sigma-Aldrich) three times and resuspended in 6 M guanidine (Sigma-Aldrich). Protein concentrations were estimated using a bicinchoninic acid assay (Thermo Scientific). Fifty micrograms samples of proteins were further processed and digested using filter-aided sample preparation^63,64^. Peptides were measured by an 11-step multidimensional protein identification technology^65^, as described previously^14,63^. Tandem mass spectrometry spectra from each soil sample were searched using Sipros Ensemble^66^ against a matched protein database constructed from the metagenome of that sample. Raw search results were filtered to achieve 1% FDR at the peptide level, estimated by the target–decoy approach^67^. Proteins were inferred from identified peptides using a parsimony rule^68^. A minimum of one unique peptide was required for each identified protein or protein group. FDR at the protein level of each sample was below 3%.

Proteins confidently identified in each sample were annotated using hmmsearch against the dbCAN v6 HMM database with default parameters^69^. The results were filtered to remove hits with an e-value ≥ 1 × 10^−14^ and HMM coverage ≤0.35. For CAZy domains overlapping the same region of sequence, the domain with the lower e-value was selected. Carbon and nitrogen metabolic functions were annotated by using HMMER3 against an in house HMM database built from the Kyoto Encyclopedia of Genes and Genomes (KEGG) orthology groups (see Genome metabolic annotation section for further information). For methanol dehydrogenase (xoxF) and CO dehydrogenase (coxL) genes we determined subgroup membership using initial HMM placement and subsequent phylogenetic classification as described below in Genome metabolic annotation. All annotations were concatenated and a protein that received confident annotations from more than one database was assigned the annotation with the highest e-value score.

To enable comparison across samples, proteins from each sample were clustered into their assigned functional orthology groups, and the spectral counts for proteins in the same sample with the same functional assignment were summed. Before performing further statistical analysis, functions that were present in fewer than five samples were removed from the analysis. Subsequently, the remaining functions were ranked in each sample based on the total spectral counts assigned to a function, and the mean rank for a function was calculated across samples.

To look at over-representation of KEGG functions in our proteomic data set, we compared the total number of proteins in our data set annotated with a KEGG KO to the number of proteins with that KEGG KO in the KEGG database. Over-enrichment was determined using the hypergeometric test, implemented as the phyper function in R. All hypergeometric *P* values were then corrected for multiple testing using FDR.

### Genome binning, curation and dereplication

Metagenome assemblies were binned into draft genomes using a dereplication and aggregation strategy using the output of multiple metagenomic binning programs. Reads from all 60 samples were mapped to contigs >2 kbp using Bowtie2, and a differential coverage profile for each contig across all samples was used as input for the following differential coverage binners: ABAWCA, ABAWACA2, MaxBin2, CONCOCT and MetaBAT^70–72^. The algorithm DasTool^73^ was then used to select the highest quality bins from each metagenome assembly. Bins were then manually inspected through the ggKbase web server and contigs with phylogenetic signatures that significantly deviated from bin taxonomy were removed. Bin completeness and contamination were then assessed using CheckM^74^ and bins were filtered based on an established metric of ≥70% completeness^21^. Bins were then dereplicated across samples by matching the rpS3 containing contigs in each SG to their respective bins. The bin with the highest completeness and lowest contamination associated with each SG was then selected to be a representative of that SG. This resulted in 896 bins associated with SGs (Supplementary Table 5). Scaffolding errors in the dereplicated bin set were corrected as previously described^21^. Gene loci in these bins were then recalled using Prodigal in single genome mode. Error corrected bins were assessed again for completeness and contamination with CheckM, and 793 bins passed the criteria of ≥70% completeness and <10% contamination that we required for inclusion in our metabolic analysis.

### Genome phylogenetic classification

The taxonomy of microorganisms represented by the 896 dereplicated bins was determined using the combination of a concatenated ribosomal protein tree, rpS3 protein tree and 16S rRNA gene sequences binned with genomes. For the ribosomal protein tree we searched each genome for 15 ribosomal proteins (L2, L3, L4, L5, L6, L14, L15, L16, L18, L22, L24, S3, S8, S17, S19) using USEARCH against a database of ribosomal proteins from ref. ^51^. If ribosomal proteins in a genome were not found in a contiguous block, we manually checked if any of the ribosomal protein containing contigs represented a contaminating sequence. A total of 852 genomes containing eight or more ribosomal proteins were then included in the analysis. Ribosomal protein sequences were individually combined with reference ribosomal protein sequences from ref. ^51^ and selected sequences from ref. ^75^. Sequences were then individually aligned using MAFFT. The resulting alignments were stripped of columns containing >95% gap positions. Individual stripped alignments were concatenated and a phylogenetic tree was constructed using RAxML v8.2.10^76^ on the CIPRES Science Gateway{Miller:vv}. RAxML was called as follows: raxmlHPC-HYBRID -s input -N autoMRE -n result -f a -p 12345 -x 12345 -m PROTCATLG. Genomes were then manually assigned phylum- and class-level lineage information based on their position relative to reference sequences in the tree. See also Supplementary Fig. 4 and Supplementary Data 5–7. In the case where a genome was not included in the ribosomal protein tree, its taxonomy assigned by the rpS3 tree was inherited. For acidobacterial genomes, class-level assignments were made by a combination of ribosomal protein tree assignments and predicted 16S rRNA gene sequence taxonomy. See also Supplementary Fig. 5.

16S rRNA gene sequences identified within metagenome bins (see above) were aligned using SINA v1.2.11 implemented on the SILVA ACT web portal^77^. Sequences were aligned against the global SILVA alignment for SSU rRNA genes, and sequences with an alignment identity ≥70% were then classified using the least common ancestor method based on taxonomies in SILVA. See also Supplementary Table 6.

### Genome metabolic annotation

For the 793 bins passing completeness and contamination criteria, carbohydrate active enzymes (CAZy) were annotated using hmmsearch against the dbCAN v6 HMM database with default parameters^69^. The results were filtered to remove hits with an e-value ≥ 1 × 10^−14^ and HMM coverage of ≤0.35. For CAZy domains overlapping the same region of sequence, the domain with the lower e-value was selected. Carbon and nitrogen metabolic functions were annotated by using HMMER3 against an in house HMM database built from KEGG orthology groups (KOs). Briefly, all KEGG database proteins with KOs were compared with all-v-all global similarity search using USEARCH. MCL was then used to sub-cluster KOs (inflation_value = 1.1). Each sub-cluster was aligned using MAFFT, and HMMs were constructed from sub-cluster alignments. HMMs were then scored against all KEGG sequences with KOs and a score threshold was set for each HMM at the score of the highest scoring hit outside of that HMMs sub-cluster. Access to the proprietary KEGG database was secured via contract, so only our procedure to profile them can be made public.

For methanol dehydrogenase (xoxF), CO dehydrogenase (coxL) and nitrite reductase (nirK) we constructed individual phylogenetic trees to discriminate homologous, but functionally distinct, proteins that can be identified by HMM search alone. XoxF sequences were initially identified in genomes using a custom HMM for PQQ-binding alcohol dehydrogenases^21^. Angelo sequences were combined with reference sequences from refs. ^33,78^ and aligned using MAFFT. A phylogenetic tree was constructed using FastTree (Supplementary Fig. 7 and Supplementary Data 12) and xoxF sequences were manually discriminated from mxaF and general ADH sequences by their position relative to reference sequences in the tree.

Putative coxL sequences were identified by KEGG HMM hits to K03520. Angelo hits were combined with reference sequences from ref. ^9^, and aligned using MAFFT. A phylogenetic tree was constructed using FastTree (Supplementary Fig. 8 and Supplementary Data 13) and coxL-typeI sequences were manually identified by a known sequence motif ‘AYRCSFR’^22^ and their position relative to reference sequences in the tree.

Putative nirK sequences were identified by KEGG HMM hits to K00368. Angelo hits were combined with reference sequences from ref. ^79^ and aligned using MAFFT. A phylogenetic tree was constructed using FastTree (Supplementary Fig. 9 and Supplementary Data 14), and true NirK sequences were manually identified by the presence of properly aligned catalytic residues and their position relative to reference sequences in the tree.

C_1_ carbon and inorganic nitrogen metabolism were assessed by looking at a specific set of 28 targeted functions. For further information on annotation criteria and functional assignments to genomes see Supplementary Tables 9–13.

### Depth and treatment enrichment analysis

We first assessed the differences between estimated completeness and contamination for the sets of genomes that would be compared when testing for enrichment (Supplementary Fig. 14a,b and Supplementary Table 19). For each condition tested (Depth, Treatment – 20 cm and Treatment – 40 cm), the estimated genome completeness and contamination values across the three response groups (Increase, Decrease and Neither) were initially tested for significant differences using the Kruskal–Wallis rank sum test^80^, implemented as the kruskal.test function in R. The Kruskal–Wallis *P* values were corrected for multiple testing using FDR, and post hoc testing between specific response groups was undertaken for FDR ≤ 0.1. Post hoc testing was carried between all pairs of response groups in a condition using the Wilcoxon rank sum test implemented as the pairwise.wilcox.test function in R^81,82^, and corrected for multiple testing using FDR. An FDR ≤ 0.05 in post hoc testing was considered significant.

Significant enrichments of phylum level lineages and 29 targeted metabolic functions were assessed between genome response groups in each condition using Fisher’s exact test^83^ followed by post hoc testing with a permutation analysis (Supplementary Tables 9, 10 and 14, 15). We first removed all metabolic functions or phyla from the analysis that were not present in both the increased or decreased genome groups from a condition. Then, for each factor tested across the three conditions (Depth, Treatment – 20 cm and Treatment – 40 cm) counts in the three genome response groups (Increase, Decrease and Neither) were first compared using Fisher’s exact test^83^ on a 2 × 3 contingency table, implemented as the fisher.test function in R. Fisher test *P* values were corrected using FDR, and post hoc testing was carried out on functions or phylum categories with FDR ≤ 0.1. Post hoc testing was then only conducted on groups of genomes that increased or decreased with respect to a condition. We carried out post hoc testing using a permutation test implemented as a custom R function to reflect the underlying frequency distribution of the phylum or functional gene being tested across all 793 bins that were analysed (see Code availability statement). Briefly, the counts of each function or phylum in the increased or decreased sets of genomes were randomly resampled without replacement 10,000 times from all 793 genomes. The absolute value of the difference between the fraction of counts of a phylum or function over the total number of genomes in the respective increased or decreased set was then calculated. *P* values were calculated as the number of absolute fractional differences in the permuted set that exceeded the observed fractional difference divided by 10,000 samples. *P* values were corrected using FDR, and FDR values ≤0.05 were considered significant.

CAZy enzyme Shannon and Simpson diversity for genomes was quantified using the diversity function in the R vegan package^55^. Unique counts of CAZy enzymes per genome were quantified with the specnumber function in the R vegan package. For each diversity metric calculated across the three conditions (Depth, Treatment – 20 cm and Treatment – 40 cm), the three genome response groups (Increase, Decrease and Neither) were first compared using the Kruskal–Wallis rank sum test^80^, implemented as the kruskal.test function in R. Kruskal–Wallis *P* values were corrected using FDR, and post hoc testing between groups of genomes that increased and decreased with respect to a condition was conducted for all FDR ≤ 0.1. Post hoc testing was carried out using the Wilcoxon rank-sum test implemented as the wilcox.test function in R^81,82^, and corrected for multiple testing using FDR. Differences were considered significant for FDR values ≤0.05. Differential enrichment of specific CAZy classes was tested using the same procedure as for diversity metrics, with initial three category testing being performed with the Kruskal–Wallis test, subsequent post hoc testing between increased and decreased genomes being performed with the Wilcoxon rank-sum test, and multiple testing being corrected with FDR.

Feature selection of KEGG KOs that were significant predictors of a genome having increased or decreased abundance with depth was undertaken using the random forest-based method Boruta, implemented in R^84^. Briefly, KO profiles from genomes showing a depth response were subset and KOs present in ≤5 genomes of the total set were removed from the data. Due to the significantly different number of genomes that increase and decrease with depth case weights were applied based on the ratio of increasing to decreasing genomes (Decrease = 2.184, Increase = 1). Brouta was then called with the following options: Depth_Change ~., doTrace = 2, maxRuns = 500, num.trees = 7,500, case.weights = cs_wts. All features confirmed as significant predictors by Boruta were then individually tested for differential abundance between the genome sets that decreased and increased with depth using the Wilcoxon rank-sum test. Wilcoxon *P* values were FDR corrected, and KOs with FDR ≤ 0.05 were considered significant. For full Boruta output see Supplementary Table 18.

### Functional gene co-occurrence and correlation analysis

The co-occurrence overview of 29 targeted carbon and nitrogen turnover functions annotated in our 793 genomes (Supplementary Fig. 12 and Supplementary Table 10) was produced using the pheatmap function in R^82^. Clustering was performed using binary distance and Ward hierarchical grouping^85^. Correlations and correlation *P* values for the co-occurrence of functions across all genomes (Supplementary Fig. 13) were calculated using Spearman rank correlation implemented with the rcorr function in R^82^. All *P* values were corrected using FDR, and FDR values ≤0.05 were considered significant. Significant correlations between functional genes were plotted using the corrplot function from the corrplot package in R (https://github.com/taiyun/corrplot). Correlations were clustered using the angular order of the eigenvectors implemented in the corrplot package, and cluster groups were human defined.

### Reporting Summary

Further information on research design is available in the Nature Research Reporting Summary linked to this article.

## Online content

Any methods, additional references, Nature Research reporting summaries, source data, statements of code and data availability and associated accession codes are available at 10.1038/s41564-019-0449-y.

## Supplementary information

Supplementary Information Supplementary Results, Supplementary Figures 1–14, legends for Supplementary Tables, legends for Supplementary Datasets and Supplementary References.

Reporting Summary

Supplementary Tables Supplementary Tables 1–19.

Supplementary Dataset 1 All rpS3 centroid sequences.

Supplementary Dataset 2 All rpS3 containing longest scaffolds for species groups.

Supplementary Dataset 3 Full rpS3 protein tree with reference sequence.

Supplementary Dataset 4 rpS3 protein tree with only sequences from our study.

Supplementary Dataset 5 Full concatenated ribosomal protein tree with references.

Supplementary Dataset 6 Full concatenated ribosomal protein alignment for ribosomal protein tree.

Supplementary Dataset 7 Individual ribosomal protein sequence sets.

Supplementary Dataset 8 All 16S sequences identified in our study.

Supplementary Dataset 9 Full proteomics protein count data.

Supplementary Dataset 10 All KEGG HMM-based annotations for metabolically analysed genomes.

Supplementary Dataset 11 All filtered dbCAN HMM hits for metabolically analysed genomes.

Supplementary Dataset 12 Full pqq-alcohol dehydrogenase protein tree.

Supplementary Dataset 13 Full CoxL carbon monoxide dehydrogenase-like protein tree.

Supplementary Dataset 14 Full NirK nitrite reductase protein tree.

## Data Availability

Genomic data, including curated genomes and raw sequencing reads, are available under NCBI BioProject accession no. PRJNA449266. Proteomic data are available through the ProteomeXchange Consortium via the PRIDE partner repository with identifier PXD013110.

## Code availability

Code used in the analysis for this Article are available at the following GitHub repository: https://github.com/SDmetagenomics/Angelo2019_Paper
