# Supplementary material for: Mediterranean grassland soil C–N compound turnover is dependent on rainfall and depth, and is mediated by genomically divergent microorganisms
Source: Nat Microbiol. 2019 May 20;4(8):1356–67. doi: 10.1038/s41564-019-0449-y (PMC6784897; doi:10.1038/s41564-019-0449-y)
Supplement: Supplementary file 1 — Supplementary Results, Supplementary Figures 1–14, legends for Supplementary Tables, legends for Supplementary Datasets and Supplementary References. [file 41564_2019_449_MOESM1_ESM.pdf]

nature microbiology

SUPPLEMENTARY INFORMATION

https://doi.org/10.1038/s41564-019-0449-y

In the format provided by the authors and unedited.

Mediterranean grassland soil C–N compound turnover is dependent on rainfall and depth, and is mediated by genomically divergent microorganisms

Spencer Diamond[1], Peter F. Andeer[2], Zhou Li[3], Alexander Crits-Christoph[4], David Burstein[1,7], Karthik Anantharaman[1,8], Katherine R. Lane[1], Brian C. Thomas[1], Chongle Pan[3,9], Trent R. Northen[2,5] and Jillian F. Banfield[1,6]*

[1]Department of Earth and Planetary Science, University of California, Berkeley, Berkeley, CA, USA. [2]Environmental Genomics and Systems Biology Division, Lawrence Berkeley National Laboratory, Berkeley, CA, USA. [3]Oak Ridge National Laboratory, Oak Ridge, TN, USA. [4]Department of Plant and Microbial Biology, University of California, Berkeley, Berkeley, CA, USA. [5]Joint Genome Institute, Lawrence Berkeley National Laboratory, Walnut Creek, CA, USA. [6]Department of Environmental Science, Policy, and Management, University of California, Berkeley, Berkeley, CA, USA. [7]Present address: School of Molecular Cell Biology and Biotechnology, Tel Aviv University, Tel Aviv, Israel. [8]Present address: Department of Bacteriology, University of Wisconsin, Madison, WI, USA. [9]Present address: School of Computer Science and Department of Microbiology and Plant Biology, University of Oklahoma, Norman, OK, USA. *e-mail: jbanfield@berkeley.edu

Supplementary Materials

for

Mediterranean grassland soil C-N compound turnover is depth stratified, rainfall dependent, and is mediated by genomically divergent microorganisms

Supplementary Results

Species richness census indicates robust tracking, and deep sampling, of microorganisms from a highly complex community. Our SG survey indicated that these soils are heterogeneous with a high prevalence of relatively low abundant organisms, as 2120 (63.7%) SGs were only assembled from one of our 60 metagenomic samples. However, by cross mapping reads from all 60 samples back to our representative SG sequences, we could detect and track the presence of a SG in a sample even when it was below the ~2x coverage threshold required for assembly (Supplementary Fig. 2a-b). We found that the 2120 sequences reconstructed in only one sample could be confidently detected on average in 31±18 samples at low abundance (Supplementary Table 2 and Supplementary Fig. 2b).

The iChao2 metric and a permuted collectors curve were used to estimate species richness and assess the impact of possible further sampling on additional SG recovery, respectively (Supplementary Fig. 2c-e). The iChao2 metric estimated total species richness at 9183 organisms (95% CI: 8641 - 9780 organisms). Thus, our SGs represent 34% - 38% of the total organisms present at this site by number. Our collectors curve indicated that we did not saturate SG recovery, however the slope of the recovery curve indicates that we have saturated high level recovery and further sampling would only yield around 30 additional SGs per additional sample (Supplementary Fig. 2d-e).

Metabolic functions in our proteome samples show strong enrichment relative to a null background set. To enable comparison across samples, proteins from each sample were assigned and clustered into functional orthology groups (Supplementary Tables 7-8 and Supplementary Data 9). We observed that the top 50 functions account for 57% of annotated proteins by abundance, suggesting that a small set of metabolic enzymes may be particularly

important in this system (Supplementary Fig. 6). To determine if the proteins identified in our set represent particularly abundant groups relative to a null background, we tested if KEGG functions we observed were enriched in our sample by comparing their frequency in our dataset against the frequency of these functions in the full KEGG database. Our findings indicate that 82% of the KEGG functions we observed were statistically enriched (FDR ≤ 0.05; one-sided hypergeometric test) relative to their background frequency in the KEGG database (Supplementary Fig. 6 and Supplementary Table 8). We note all of the top 50 functions were significantly enriched in our dataset, with the exception of the XoxF and CoxL enzymes that were not assigned to KEGG orthology groups (Methods). While we observe strong enrichment results for many of the proteins in our study, we caution that sampling depth for proteomics is significantly less than for metagenomic analysis, and that the quantity of observed proteins can be complicated by protein stability and the recalcitrance of specific protein groups (i.e. membrane proteins) to proteomics extraction methods.

Complementary C1 metabolic functions co-occur in genomes. We looked at the co-occurrence of 29 targeted carbon and nitrogen transformation functions across our 793 genomes (Supplementary Fig. 12). Generally, we see that while many genomes in this system have some C1 metabolic potential, there are distinct clusters where genomes encode multiple small compound degradation, nitrogen turnover, and C1 metabolic functions. Phylogenetically the genomes encoding larger repertoires of the analyzed functions tend to fall within the proteobacterial, acidobacterial, and rokubacterial groups.

To interrogate which functions tend to be associated in genomes, we performed a co-occurrence correlation analysis across all 793 genomes for the 29 functional genes annotated (Supplementary Fig. 13). The results indicate roughly 2 correlated clusters of genes, with correlations generally existing between functions for small molecule degradation and functions

for processing the downstream products of small molecule degradation. We see in cluster 1 that, unlike nitrogen turnover processes (Fig. 3B), the functions for sequential steps in C1 carbon processing show significant levels of co-occurrence. Cluster 1 also includes mauAB which can liberate formaldehyde from methylamine. In a similar connection of small molecule degradation to downstream turnover, Cluster 2 indicates positive association between three degradative processes that release inorganic nitrogen and three inorganic nitrogen turnover functions. Interestingly, we note that there was a significant negative association between amo_pmo and xoxF. This would suggest that particulate monooxygenases in this system are not involved in methanotrophy, and more likely function in ammonia oxidation.

Individual CAZy enzyme classes show depth and treatment dependent changes. In addition to quantifying and comparing CAZy enzyme diversity between genomes that change in abundance with depth and extended rainfall treatment, we also tested if specific CAZy enzyme functional classes were enriched in these changing groups (Supplementary Table 17). Between the genomes that changed in abundance across depth we found 32 enzymes that were differentially enriched between genome groups that increased and decreased with depth. CAZy enzymes from 29 different classes were statistically enriched in genomes more abundant in shallow soil, and 29% of these enzymes are known to use forms of starch as a substrate (Supplementary Table 17). Relative to shallow soil, only three CAZy classes were enriched in genomes that were more abundant at deeper depth (Supplementary Table 17). In 10-20 cm samples, 14 CAZy enzyme classes showed differential enrichment between the groups of genomes where abundance increased and decreased in response to extended rainfall. However, both genome groups each had 7 enzymes that were differentially enriched, and there was no clear pattern of compound utilization in either group. Alternatively, in 30-40 cm samples, there were 23 CAZy classes enriched in genomes whose abundance increased in response to

extended rainfall and only one CAZy class enriched in genomes whose abundance decreased. The majority of the 23 enzymes that were enriched in genomes that increased in abundance at 30-40 cm have predicted activity on pectin and hemicellulose (Supplementary Table 17). Thus, there are generally more, and more diverse, CAZy enzymes in genomes that are more abundant closer to the surface, and many of those enzymes act on simple carbohydrates such as starch. With extended rainfall treatment, the primary difference occurs at 30-40 cm depth, and consists of an increase in enzymes that act on more complex and recalcitrant plant polymers.

Additional metabolic features with depth dependent abundance patterns were identified by a machine learning approach. Using a random forest based feature selection approach (Methods) we identified KEGG functions that had a significant association with genomes that either increased or decreased in abundance with depth. We identified 131 and 280 KEGG functions that were statistically enriched in genomes that increased or decreased in abundance with depth, respectively (Supplementary Table 18). Organisms more abundant at depth were significantly enriched in PII nitrogen regulatory proteins and organisms more abundant at shallower depth had significantly higher proportions of small molecule dehydrogenases active on xanthines and succinate (Supplementary Table 18). Generally, these results indicate simple carbon metabolism is more common in organisms closer to the surface and inorganic nitrogen metabolism is more common in organisms more abundant at deeper depth.

Supplementary Figures

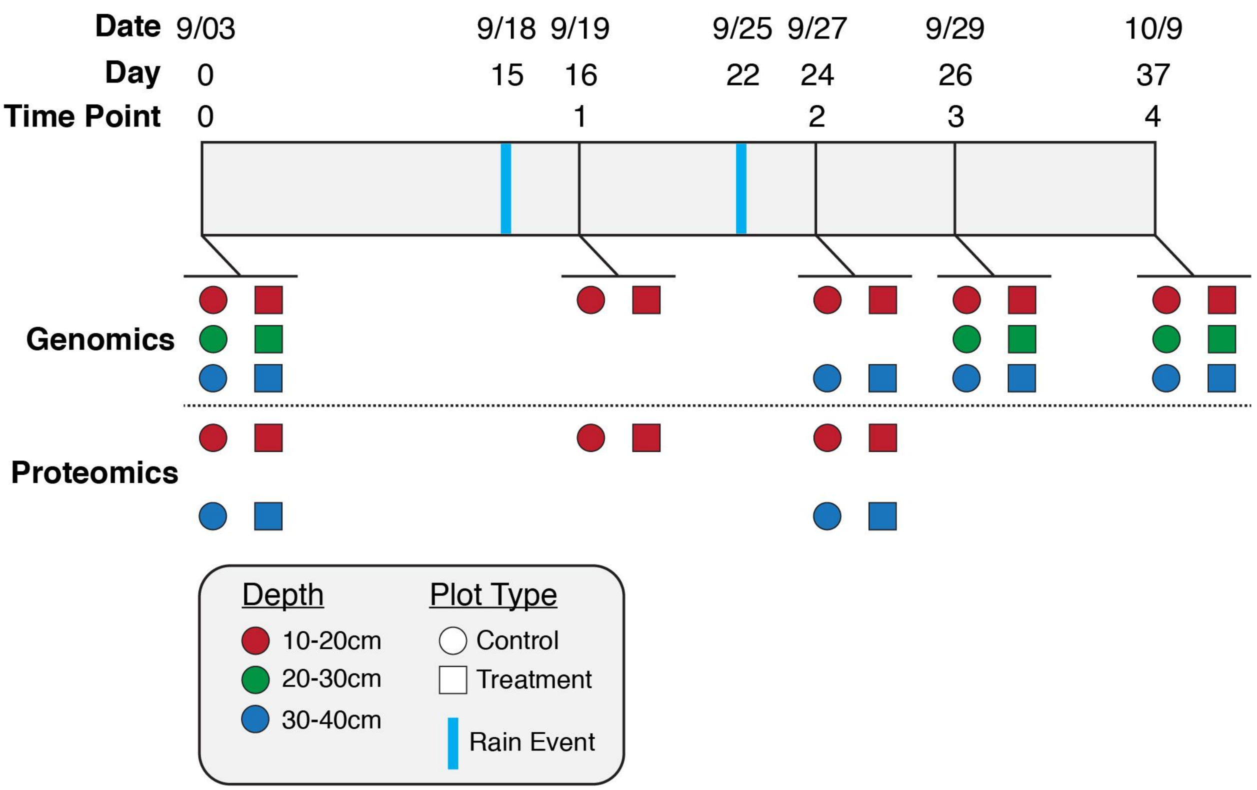

Supplementary Figure 1 l Sampling scheme for metagenomics and proteomics samples. Plot shows the dates samples were taken in 2013 as well as dates of rain events that occurred at the site. Time point corresponds with the time points depicted in Figure 1b. The presence of a dot in the genomics or proteomics row indicates a sample was taken on that date of the given type. Also, see Supplementary Table 1.

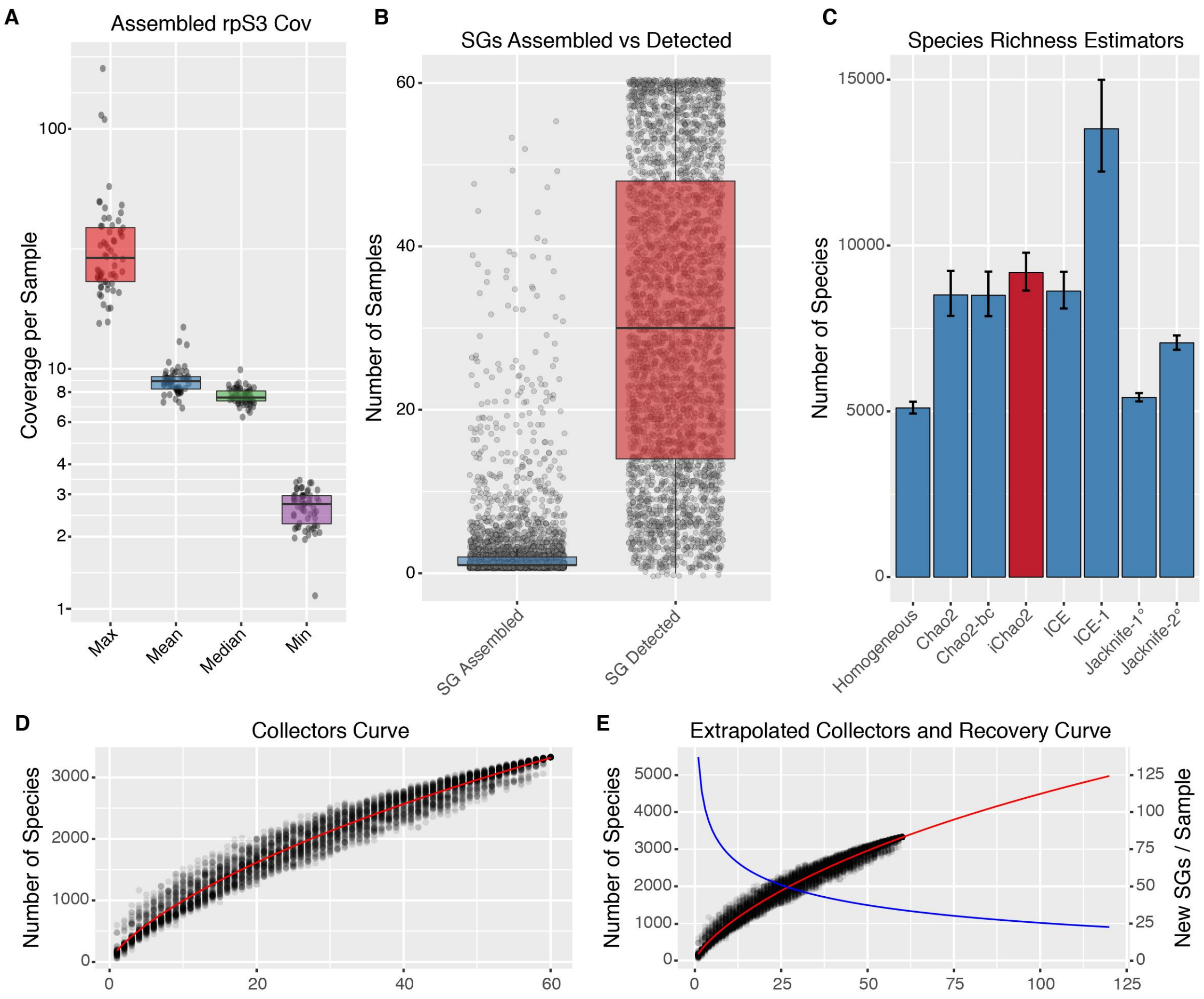

A
Assembled rpS3 Cov
Coverage per Sample
Max
Mean
Median
Min
B
SGs Assembled vs Detected
Number of Samples
SG Assembled
SG Detected
C
Species Richness Estimators
Number of Species
Homogeneous
Chao2
Chao2-bc
iChao2
ICE
ICE-1
Jacknife-1°
Jacknife-2°
D
Collectors Curve
Number of Species
E
Extrapolated Collectors and Recovery Curve
Number of Species
New SGs / Sample

Supplementary Figure 2 | rpS3 Mapping, estimated species richness, and collectors curves. (A) Summary coverage statistics for rpS3 sequences assembled in each sample. Each point represents one of our 60 samples. Shaded box area indicates 1st to 3rd quartile range for data, and black line indicates median. **(B)** Comparison showing the number of samples, out of 60 total samples, an rpS3 sequence was assembled in vs the number of samples it could be detected in (at least 2 mapped reads >99% ID; n = 3325 independent rpS3 sequences). Shaded box area indicates 1st to 3rd quartile range for data, black line indicates median, and whiskers encompass 1.5*interquartile range. **(C)** Species richness estimators calculated with the SpadeR package based on the rpS3 counts table (n = 3325 independent rpS3 sequences; Supplementary Table 3). iChao2 metric is shown in red and used as the primary estimator in the paper. Bars indicate the mean of the estimate and error bars depict the 95% confidence interval. **(D)** Permuted collectors curve for random selections of 1-60 samples. Black dots indicate number of unique species recovered for one permutation. Red curve is the lomolino fit to the points. **(E)** Permuted collectors curve extrapolated to 120 samples. Red curve is lomolino fit and blue curve is the estimated slope of the fit representing the number of additional species expected to be recovered at the given number of samples.

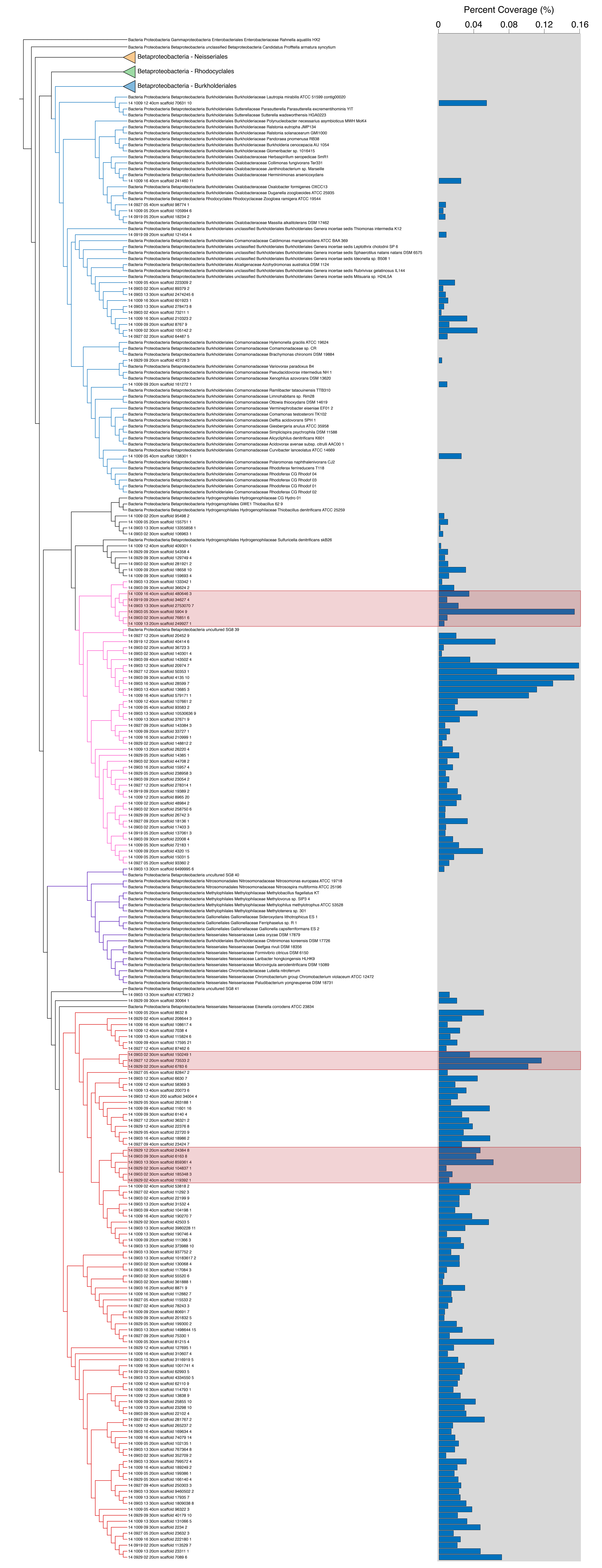
Percent Coverage (%)
0
0.04
0.08
0.12
0.16
Betaproteobacteria - Neisseriales
Betaproteobacteria - Rhodocyclales
Betaproteobacteria - Burkholderiales

Supplementary Figure 3 l Sample of rpS3 tree showcasing variance in abundance of closely related organisms. A representative section of the full rpS3 protein tree, using Betaproteobacteria as an example. The full tree was constructed using FastTree from an alignment of 5,649 rpS3 protein sequences (3,325 identified in our data and 2,324 reference sequences). This subset showcases instances where variability in abundance between species groups (SGs) that are phylogenetically similar is large (Red Boxes). rpS3 SGs are named by the sequence ID for the centroid rpS3 within their sequence cluster. The sequence IDs can be cross referenced to SG names in Supplementary Table 2. Nodes were collapsed for roughly class level lineages of Betaproteobacteria where no sequences from our study were found. Branches of the tree are colored by their class level phylogeny from top to bottom as follows: Burkholderiales - Light Blue; ANG-BPRX1 - Pink; Nitrosomonadales - Purple; ANG-BPRX2 - Red. The bar plot in line with the tree gives the coverage of each SG across our samples as a percent of total mapped coverage. The full rpS3 tree can be found as Supplementary Data 3.

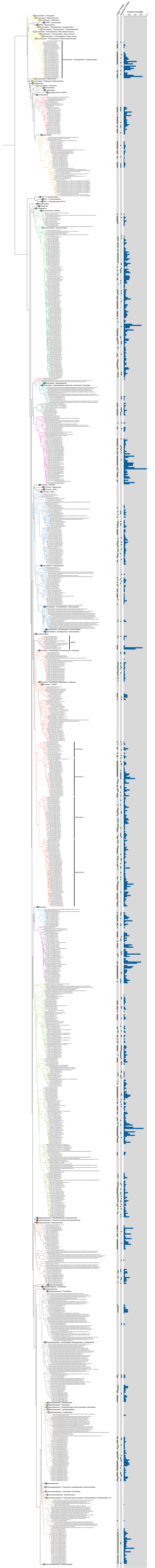
Depth Change
Treatment Change
Percent Coverage
0
0.25
0.50
0.75
1.0
Euryarchaeota - Archaeoglobi
Euryarchaeota - Methanomicrobia
Euryarchaeota - Halobacteria
DPANN - Pacearchaeota
DPANN - Woesarchaeota
Euryarchaeota - Thermoplasmata - Aciduliprofundum
Euryarchaeota - Thermoplasmata - Thermoplasmatales
Euryarchaeota - Thermoplasmata - Marine Benthic Group D
Euryarchaeota - Thermoplasmata - Marine Group III
Euryarchaeota - Thermoplasmata - Marine Group II
Euryarchaeota - Thermoplasmata - Methanomassiliicoccales
Euryarchaeota - Thermoplasmata - Angelarchaeales
Euryarchaeota - Methanococci
Euryarchaeota - Methanopyri; Methanobacteria
Hadesarchaea
Euryarchaeota - Thermococci
Thorarchaeota
Lokiarchaeota
Candidate Division YNPFFA
Crenarchaeota
Aigarchaeota

Supplementary Figure 4 l Full rp15 species tree. Phylogenetic tree constructed using RaxML from a concatenated alignment of 15 co-located ribosomal proteins (L2, L3, L4, L5, L6, L14, L15, L16, L18, L24, S3, S8, S17, S19). The tree includes 1,916 genomes where 8 or more ribosomal proteins were identified (852 genomes identified in our study and 1,064 references). Nodes in the tree were collapsed at roughly class or order level if they did not contain genomes from our study. Phylum level clades are colored as in Fig. 3. RAxML bootstrap values are present on the nodes where bootstrap support > 90 (142 bootstrap replicates). Organisms are named based on their bin names found in Supplementary Table 5. Panels on the right of the tree indicate if an organism exhibited a change with depth or treatment, and the abundance of an organism based on the coverage of its rpS3 contig. Also, see Figure 2 and Supplementary Data 5-7

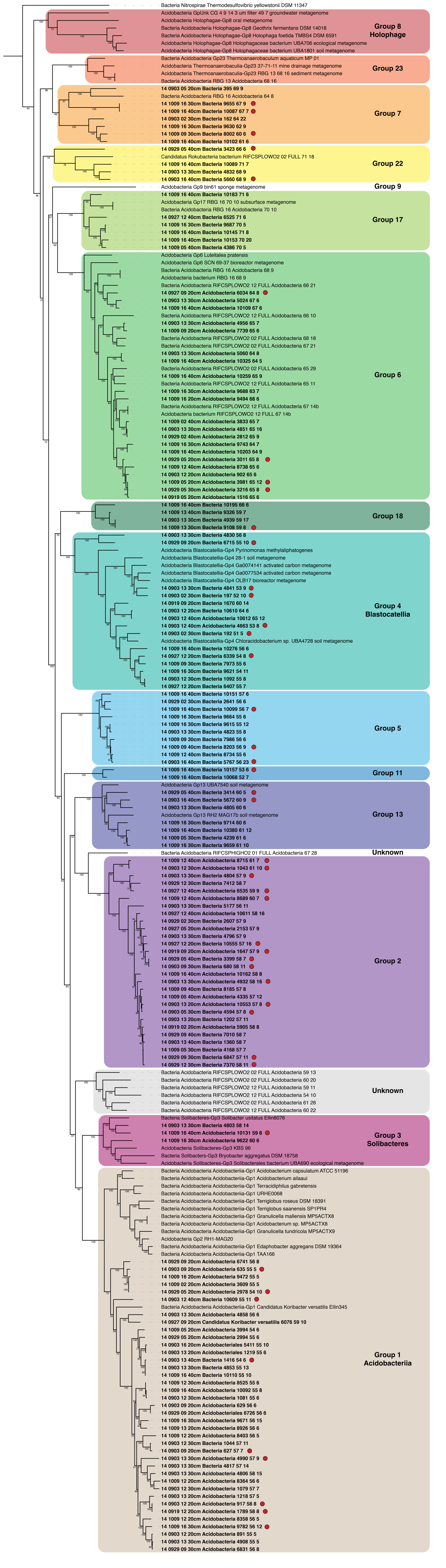

Bacteria Nitrospirae Thermodesulfovibrio yellowstonii DSM 11347
Acidobacteria GpUnk CG 4 9 14 3 um filter 49 7 groundwater metagenome
Acidobacteria Holophagae-Gp8 oral metagenome
Bacteria Acidobacteria Holophagae-Gp8 Geothrix fermentans DSM 14018
Bacteria Acidobacteria Holophagae-Gp8 Holophaga foetida TMBS4 DSM 6591
Bacteria Acidobacteria Holophagae-Gp8 Holophagaceae bacterium UBA706 ecological metagenome
Acidobacteria Holophagae-Gp8 Holophagaceae bacterium UBA1801 soil metagenome
Group 8
Holophage
Bacteria Acidobacteria Gp23 Thermoanaerobaculum aquaticum MP 01
Acidobacteria Thermoanaerobaculia-Gp23 37-71-11 mine drainage metagenome
Acidobacteria Thermoanaerobaculia-Gp23 RBG 13 68 16 sediment metagenome
Bacteria Acidobacteria RBG 13 Acidobacteria 68 16
Group 23
14 0903 05 20cm Bacteria 395 69 9
Bacteria Acidobacteria RBG 16 Acidobacteria 64 8
14 1009 16 30cm Bacteria 9655 67 9
14 1009 16 40cm Bacteria 10087 67 7
14 0903 02 30cm Bacteria 162 64 22
14 1009 16 30cm Bacteria 9630 62 9
14 1009 09 30cm Bacteria 8002 60 6
14 1009 16 40cm Bacteria 10102 61 6
Group 7
14 0929 05 40cm Bacteria 3423 66 6
Candidatus Rokubacteria bacterium RIFCSPLOWO2 02 FULL 71 18
14 1009 16 40cm Bacteria 10089 71 7
14 0903 13 30cm Bacteria 4832 68 9
14 1009 16 40cm Bacteria 5660 68 9
Group 22
Acidobacteria Gp9 bin61 sponge metagenome
Group 9
14 1009 16 40cm Bacteria 10183 71 6
Acidobacteria Gp17 RBG 16 70 10 subsurface metagenome
Bacteria Acidobacteria RBG 16 Acidobacteria 70 10
14 0927 12 40cm Bacteria 6525 71 6
14 1009 16 30cm Bacteria 9687 70 5
14 1009 16 40cm Bacteria 10145 71 8
14 1009 16 40cm Bacteria 10153 70 20
14 1009 05 40cm Bacteria 4386 70 5
Group 17
Acidobacteria Gp6 Luteitalea pratensis
Acidobacteria Gp6 SCN 69-37 bioreactor metagenome
Bacteria Acidobacteria RBG 16 Acidobacteria 68 9
Acidobacteria bacterium RBG 16 68 9
Bacteria Acidobacteria RIFCSPLOWO2 12 FULL Acidobacteria 66 21
14 0927 09 20cm Acidobacteria 6034 64 8
14 0903 13 30cm Acidobacteria 5024 67 6
14 1009 16 40cm Acidobacteria 10109 67 6
Bacteria Acidobacteria RIFCSPLOWO2 12 FULL Acidobacteria 66 10
14 0903 13 30cm Acidobacteria 4956 65 7
14 1009 09 20cm Acidobacteria 7739 65 6
Bacteria Acidobacteria RIFCSPLOWO2 02 FULL Acidobacteria 68 18
Bacteria Acidobacteria RIFCSPLOWO2 02 FULL Acidobacteria 67 21
14 0903 13 30cm Acidobacteria 5060 64 8
14 1009 16 40cm Acidobacteria 10325 64 5
Bacteria Acidobacteria RIFCSPLOWO2 02 FULL Acidobacteria 65 29
14 1009 16 40cm Acidobacteria 10259 65 9
Bacteria Acidobacteria RIFCSPLOWO2 12 FULL Acidobacteria 65 11
14 1009 16 30cm Acidobacteria 9688 63 7
14 1009 16 20cm Acidobacteria 9494 68 6
Bacteria Acidobacteria RIFCSPLOWO2 12 FULL Acidobacteria 67 14b
Acidobacteria bacterium RIFCSPLOWO2 12 FULL 67 14b
14 1009 02 40cm Acidobacteria 3833 65 7
14 0903 13 30cm Acidobacteria 4851 65 16
14 0929 02 40cm Acidobacteria 2812 65 9
14 1009 16 30cm Acidobacteria 9743 64 7
14 1009 16 40cm Acidobacteria 10203 64 9
14 0929 05 20cm Acidobacteria 3011 65 8
14 1009 12 40cm Acidobacteria 8738 65 6
14 0903 12 20cm Acidobacteria 902 65 6
14 1009 05 20cm Acidobacteria 3981 65 12
14 0929 05 30cm Acidobacteria 3216 65 8
14 0919 05 20cm Acidobacteria 1516 65 6
Group 6
14 1009 16 40cm Bacteria 10195 66 6
14 1009 13 40cm Bacteria 9326 59 7
14 0903 13 30cm Bacteria 4939 59 17
14 1009 13 30cm Bacteria 9108 59 8
Group 18
14 0903 13 30cm Bacteria 4830 56 8
14 0929 09 20cm Bacteria 6715 55 10
Acidobacteria Blastocatellia-Gp4 Pyrinomonas methylaliphatogenes
Acidobacteria Blastocatellia-Gp4 28-1 soil metagenome
Acidobacteria Blastocatellia-Gp4 Ga0074141 activated carbon metagenome
Acidobacteria Blastocatellia-Gp4 Ga0077534 activated carbon metagenome
Acidobacteria Blastocatellia-Gp4 OLB17 bioreactor metagenome
14 0903 13 30cm Bacteria 4841 53 9
14 0903 02 30cm Bacteria 197 52 10
14 0919 09 20cm Bacteria 1670 60 14
14 0903 12 20cm Bacteria 10610 64 6
14 1009 12 40cm Bacteria 10612 65 12
14 0903 12 40cm Bacteria 4663 53 8
14 0903 02 30cm Bacteria 192 51 5
Acidobacteria Blastocatellia-Gp4 Chloracidobacterium sp. UBA4728 soil metagenome
14 1009 16 40cm Bacteria 10276 56 6
14 0927 12 20cm Bacteria 6339 54 8
14 1009 09 30cm Bacteria 7973 56 5
14 1009 16 30cm Bacteria 9621 54 11
14 0903 12 30cm Bacteria 1092 55 8
14 0927 12 20cm Bacteria 6407 55 7
Group 4
Blastocatellia
14 1009 16 40cm Bacteria 10151 57 6
14 0929 02 30cm Bacteria 2641 56 6
14 1009 16 40cm Bacteria 10099 56 7
14 1009 16 30cm Bacteria 9664 55 6
14 1009 16 30cm Bacteria 9615 55 12
14 0903 13 30cm Bacteria 4823 55 8
14 1009 09 30cm Bacteria 7986 56 6
14 1009 09 40cm Bacteria 8203 56 9
14 1009 12 40cm Bacteria 8734 55 6
14 0903 16 40cm Bacteria 5767 56 23
Group 5
14 1009 16 40cm Bacteria 10157 53 6
14 1009 16 40cm Bacteria 10068 52 7
Group 11
Acidobacteria Gp13 UBA7540 soil metagenome
14 0929 05 40cm Bacteria 3414 60 5
14 0903 16 40cm Bacteria 5672 60 9
14 0903 13 30cm Bacteria 4805 60 6
Acidobacteria Gp13 RH2 MAG17b soil metagenome
14 1009 16 30cm Bacteria 9714 60 6
14 1009 16 40cm Bacteria 10380 61 12
14 1009 05 30cm Bacteria 4239 61 6
14 1009 16 30cm Bacteria 9659 61 10
Group 13
Bacteria Acidobacteria RIFCSPHIGHO2 01 FULL Acidobacteria 67 28
Unknown
14 1009 12 40cm Bacteria 8715 61 7
14 0903 12 30cm Acidobacteria 1043 61 10
14 0903 13 30cm Bacteria 4804 57 9
14 0929 12 30cm Bacteria 7412 58 7
14 0927 12 40cm Acidobacteria 6535 59 9
14 1009 12 40cm Acidobacteria 8689 60 7
14 0903 13 30cm Bacteria 5177 56 11
14 0927 12 40cm Acidobacteria 10611 58 16
14 0929 02 30cm Bacteria 2607 57 9
14 0927 05 20cm Acidobacteria 2153 57 9
14 0903 13 30cm Bacteria 4796 57 9
14 0927 12 20cm Bacteria 10555 57 16
14 0919 09 20cm Bacteria 1647 57 9
14 0929 05 40cm Bacteria 3399 58 7
14 0903 09 30cm Bacteria 680 58 11
14 1009 16 40cm Acidobacteria 10162 58 8
14 0903 13 30cm Acidobacteria 4932 58 16
14 1009 09 40cm Bacteria 8185 57 8
14 1009 05 40cm Acidobacteria 4335 57 12
14 0903 13 20cm Acidobacteria 10553 57 8
14 0903 05 30cm Bacteria 4594 57 8
14 0903 13 20cm Bacteria 1202 57 11
14 0919 02 20cm Acidobacteria 5905 58 8
14 0929 09 40cm Bacteria 7010 58 7
14 0903 13 40cm Bacteria 1360 58 7
14 1009 05 30cm Bacteria 4168 57 7
14 0929 09 30cm Bacteria 6847 57 11
14 0929 12 30cm Bacteria 7370 58 11
Group 2
Bacteria Acidobacteria RIFCSPLOWO2 02 FULL Acidobacteria 59 13
Bacteria Acidobacteria RIFCSPLOWO2 02 FULL Acidobacteria 60 20
Bacteria Acidobacteria RIFCSPLOWO2 12 FULL Acidobacteria 59 11
Bacteria Acidobacteria RIFCSPLOWO2 12 FULL Acidobacteria 54 10
Bacteria Acidobacteria RIFCSPLOWO2 02 FULL Acidobacteria 61 28
Bacteria Acidobacteria RIFCSPLOWO2 12 FULL Acidobacteria 60 22
Unknown
Bacteria Solibacteres-Gp3 Solibacter usitatus Ellin6076
14 0903 13 30cm Bacteria 4803 58 14
14 1009 16 40cm Bacteria 10131 59 8
14 1009 16 30cm Bacteria 9622 60 6
Acidobacteria Solibacteres-Gp3 KBS 96
Bacteria Solibacters-Gp3 Bryobacter aggregatus DSM 18758
Acidobacteria Solibacteres-Gp3 Solibacterales bacterium UBA690 ecological metagenome
Group 3
Solibacteres
Bacteria Acidobacteria Acidobacteriia-Gp1 Acidobacterium capsulatum ATCC 51196
Bacteria Acidobacteria Acidobacteriia-Gp1 Acidobacterium ailaaui
Bacteria Acidobacteria Acidobacteriia-Gp1 Terracidiphilus gabretensis
Bacteria Acidobacteria Acidobacteriia-Gp1 URHE0068
Bacteria Acidobacteria Acidobacteriia-Gp1 Terriglobus roseus DSM 18391
Bacteria Acidobacteria Acidobacteriia-Gp1 Terriglobus saanensis SP1PR4
Bacteria Acidobacteria Acidobacteriia-Gp1 Granulicella mallensis MP5ACTX8
Bacteria Acidobacteria Acidobacteriia-Gp1 Acidobacterium sp. MP5ACTX8
Bacteria Acidobacteria Acidobacteriia-Gp1 Granulicella tundricola MP5ACTX9
Acidobacteria Gp2 RH1-MAG20
Bacteria Acidobacteria Acidobacteriia-Gp1 Edaphobacter aggregans DSM 19364
Bacteria Acidobacteria Acidobacteriia-Gp1 TAA166
14 0929 09 20cm Acidobacteria 6741 56 8
14 0903 09 20cm Acidobacteria 635 55 5
14 1009 16 20cm Bacteria 9472 55 5
14 1009 02 20cm Acidobacteria 3609 55 5
14 0929 05 20cm Acidobacteria 2978 54 10
14 0903 12 40cm Bacteria 10609 55 11
Bacteria Acidobacteria Acidobacteriia-Gp1 Candidatus Koribacter versatilis Ellin345
14 0903 13 30cm Acidobacteria 4858 56 6
14 0927 09 20cm Candidatus Koribacter versatilis 6076 59 10
14 1009 05 20cm Acidobacteria 2994 54 6
14 0929 05 20cm Acidobacteria 2994 55 6
14 0903 16 20cm Acidobacteriales 5411 55 10
14 0903 13 20cm Acidobacteriales 1219 55 6
14 0903 13 40cm Bacteria 1416 54 6
14 0903 13 30cm Bacteria 4853 55 13
14 1009 16 40cm Bacteria 10110 55 10
14 1009 12 30cm Acidobacteria 8525 55 6
14 1009 16 40cm Acidobacteria 10092 55 8
14 0903 12 30cm Acidobacteria 1081 55 6
14 0903 09 20cm Acidobacteria 629 56 6
14 0929 09 20cm Acidobacteriales 6726 56 6
14 1009 16 30cm Acidobacteria 9671 56 15
14 1009 13 20cm Acidobacteria 8926 56 6
14 1009 12 20cm Acidobacteria 8403 56 5
14 0903 12 30cm Bacteria 1044 57 11
14 0903 09 20cm Acidobacteria 627 57 7
14 0903 13 30cm Acidobacteria 4990 57 9
14 0903 13 30cm Bacteria 4817 57 14
14 0903 13 30cm Acidobacteria 4806 58 15
14 1009 12 20cm Acidobacteria 8364 56 6
14 0903 12 30cm Acidobacteria 1079 57 7
14 0903 13 20cm Acidobacteria 1218 57 5
14 0903 12 20cm Acidobacteria 917 58 8
14 0919 12 20cm Acidobacteria 1789 58 8
14 1009 12 20cm Acidobacteria 8358 56 5
14 1009 16 30cm Acidobacteria 9782 56 12
14 0903 12 20cm Acidobacteria 891 55 5
14 0903 13 30cm Acidobacteria 4908 55 5
14 0929 09 30cm Acidobacteria 6831 56 8
Group 1
Acidobacteriia

Supplementary Figure 5 I Acidobacterial subset of rp15 tree. The acidobacterial subset of the full rp15 species tree (Supplementary Figure 4) constructed using RaxML from a concatenated alignment of 15 co-located ribosomal proteins (L2, L3, L4, L5, L6, L14, L15, L16, L18, L24, S3, S8, S17, S19). This subset includes 207 acidobacterial genomes where 8 or more ribosomal proteins were identified (145 genomes identified in our study and 62 references). Acidobacterial class level groups are indicated by colored boxes. Organism names in bold are organisms that were identified in our study. A Red dot next to an organism name indicates we recovered a 16S sequence that supports its placement in its class level clade. 16S sequences were also used to establish class names for clades containing no reference genomes of known phylogeny (i.e. Gp2). RAxML bootstrap values are present on the nodes (142 bootstrap replicates). A Nitrospira genome is present as an outgroup.

A

Gene Name	Annotation	Mean Rank	SD Rank	Annotation	FDR†
XoxF	XoxF-Tree	2.55	2.28	XoxF type Methanol Dehydrogenase	NA
SOD2	K04564	3.35	2.43	Superoxide dismutase, Fe-Mn family	0.0
livK	K01999	3.40	1.27	Branched-chain amino acid substrate-binding protein	0.0
ADH	XoxF-Tree	4.15	2.39	Alcohol dehydrogenase	NA
dppA	K02035	5.40	2.91	Peptide/nickel transport system substrate-binding protein	0.0
ycjN	K02027	7.30	3.18	Multiple sugar transport system substrate-binding protein	0.0
glpK	K00864	8.05	5.75	Glycerol kinase	0.0
K07045	K07045	12.30	6.50	COG: COG2159	0.0
gnl	K01053	12.40	5.02	Gluconolactonase	0.0
sseA	K01011	12.85	3.44	Thiosulfate/3-mercaptopyruvate sulfurtransferase	0.0
dhaK	K05878	16.50	9.23	Dihydroxyacetone kinase, N-terminal domain	0.0
CMBL	K01061	16.60	4.35	Carboxymethylenebutenolidase	0.0
CCP1	K00428	17.40	15.74	Cytochrome c peroxidase	0.0
CoxL-Hyp	CoxL-Tree	17.75	10.50	Carbon-monoxide dehydrogenase large subunit - like	NA
kynB	K07130	23.05	10.16	Arylformamidase	0.0
attM	K13075	23.10	10.01	N-acyl homoserine lactone hydrolase	0.0
iorB	K07303	24.40	6.98	Isoquinoline 1-oxidoreductase, beta subunit	0.0
CoxM	K03519	27.15	17.96	Carbon-monoxide dehydrogenase medium subunit	0.0
oppA	K15580	27.70	27.08	Oligopeptide transport system substrate-binding protein	0.0
xylF	K10543	28.00	27.79	D-xylose transport system substrate-binding protein	0.0
aglE	K10232	29.00	12.93	Alpha-glucoside transport system substrate-binding protein	0.0
CoxL-Type IIa	CoxL-Tree	29.21	18.98	Carbon-monoxide dehydrogenase large subunit – Type IIa	NA
amiF	K01455	29.90	38.70	Formamidase	$2.7e^{-224}$
rbsB	K10439	30.05	18.23	Ribose transport system substrate-binding protein	$2.7e^{-273}$
CoxL-TypeI	CoxL-TypeI	32.60	20.45	Carbon-monoxide dehydrogenase large subunit - confirmed	NA

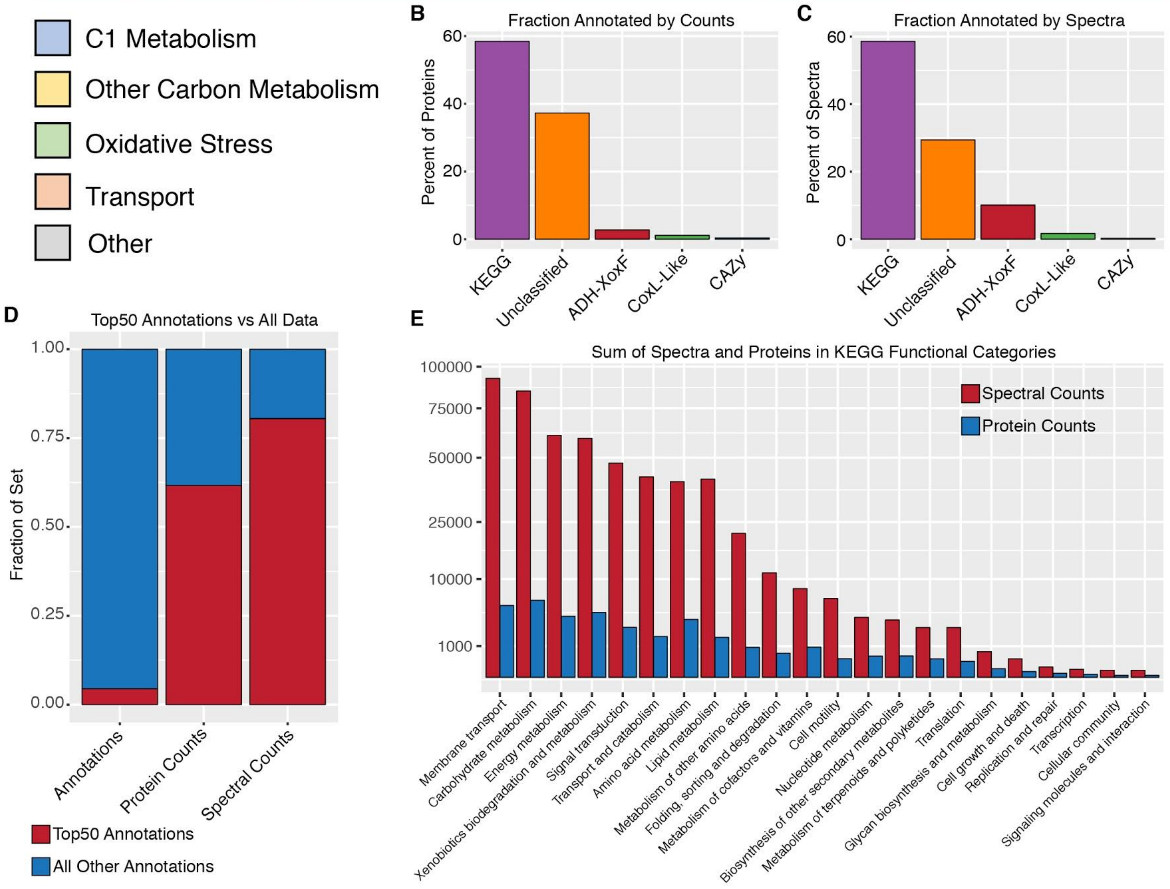

Supplementary Figure 6 l Summary of proteomics data. (A) Top 25 protein orthology groups ordered by mean rank of total spectral counts across all 20 samples. Annotations are colored by general functional class. † indicates false discovery rate (FDR) corrected significance value for enrichment of KEGG orthology groups in our sample vs. their frequency in the KEGG database (hypergeometric test) **(B)** Percent of the 55,665 proteins identified that were assigned a functional annotation by one of the major classes shown. **(C)** Percent of the total spectral counts that were assigned to a protein given a functional annotation by one of the major classes shown. **(D)** The percent the top50 annotations represent of total annotations, protein counts, and spectral counts out of the entire proteomics dataset. Red denotes the top 50 annotations while blue represents the remaining 1064 annotations. **(E)** The sum of spectra and proteins assigned to general KEGG functional categories.

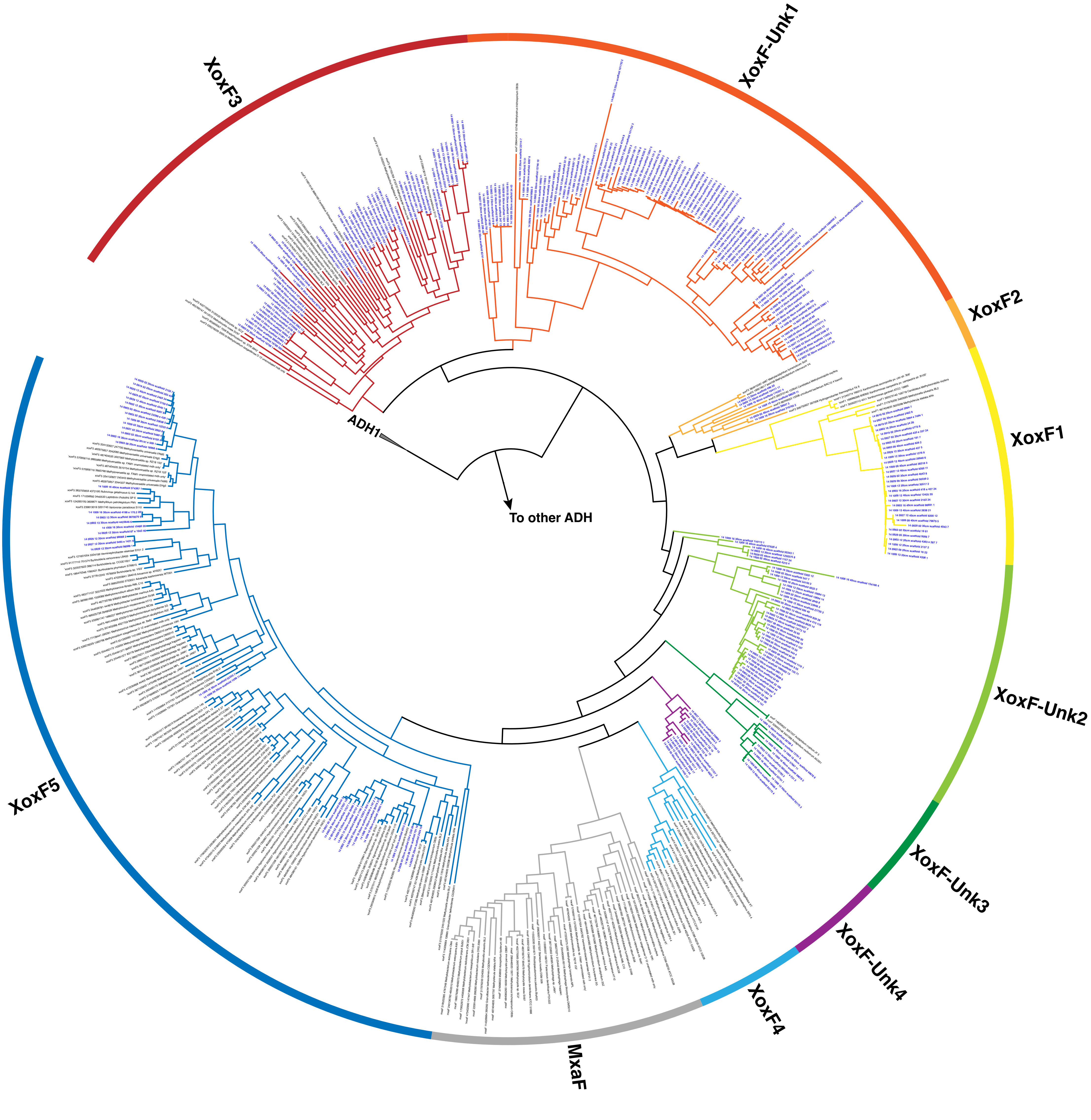

XoxF3
XoxF-Unk1
XoxF2
XoxF1
XoxF-Unk2
XoxF-Unk3
XoxF-Unk4
XoxF4
MxaF
XoxF5
ADH1
To other ADH

Supplementary Figure 7 l Subset of ADH-xoxF tree showing xoxF subgroups. Tree is a subset of Supplementary Data 12 showing the xoxF clade with the ADH1 clade as an outgroup. The full tree was constructed from an alignment of 2,218 pqq-containing alcohol dehydrogenase sequences, and this sub-tree displays 482 sequences. Clades and tree rings are colored to indicate the xoxF subtype. Names in blue text indicate sequences identified in our study. Sequences from our study are named by their scaffold and gene number. Subtypes were inferred using reference sequences from Keltjens et al. and Taubert et al. (methods). Unk subtype indicates no references were present for classification. Tree was constructed using FastTree.

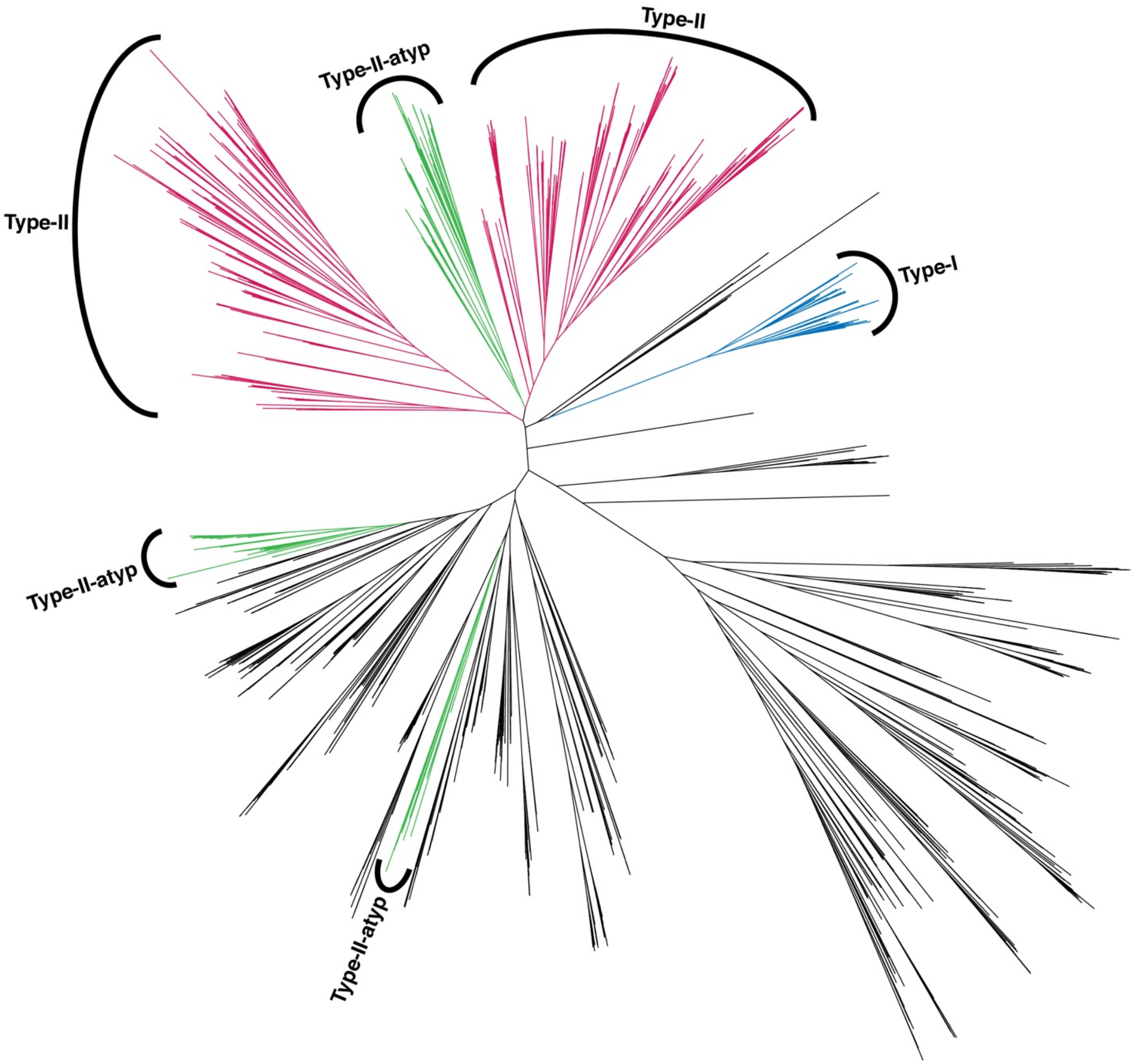

Type-II
Type-II-atyp
Type-II
Type-I
Type-II-atyp
Type-II-atyp

Supplementary Figure 8 | Unrooted coxL gene tree. This tree encompasses all 1889 coxL homologues identified by HMM search against K03520 in our study as well as sequences from Quiza et al. Labeled and colored clades indicate those where reference sequences from Quiza et al. were present (methods). Green indicates atypical coxL-TypeII sequences, magenta indicates coxL-TypeII sequences, blue indicates coxL-TypeI sequences, and black indicates unknown sequence sub-types. Tree was constructed using FastTree. For full newick tree see Supplementary Data 13.

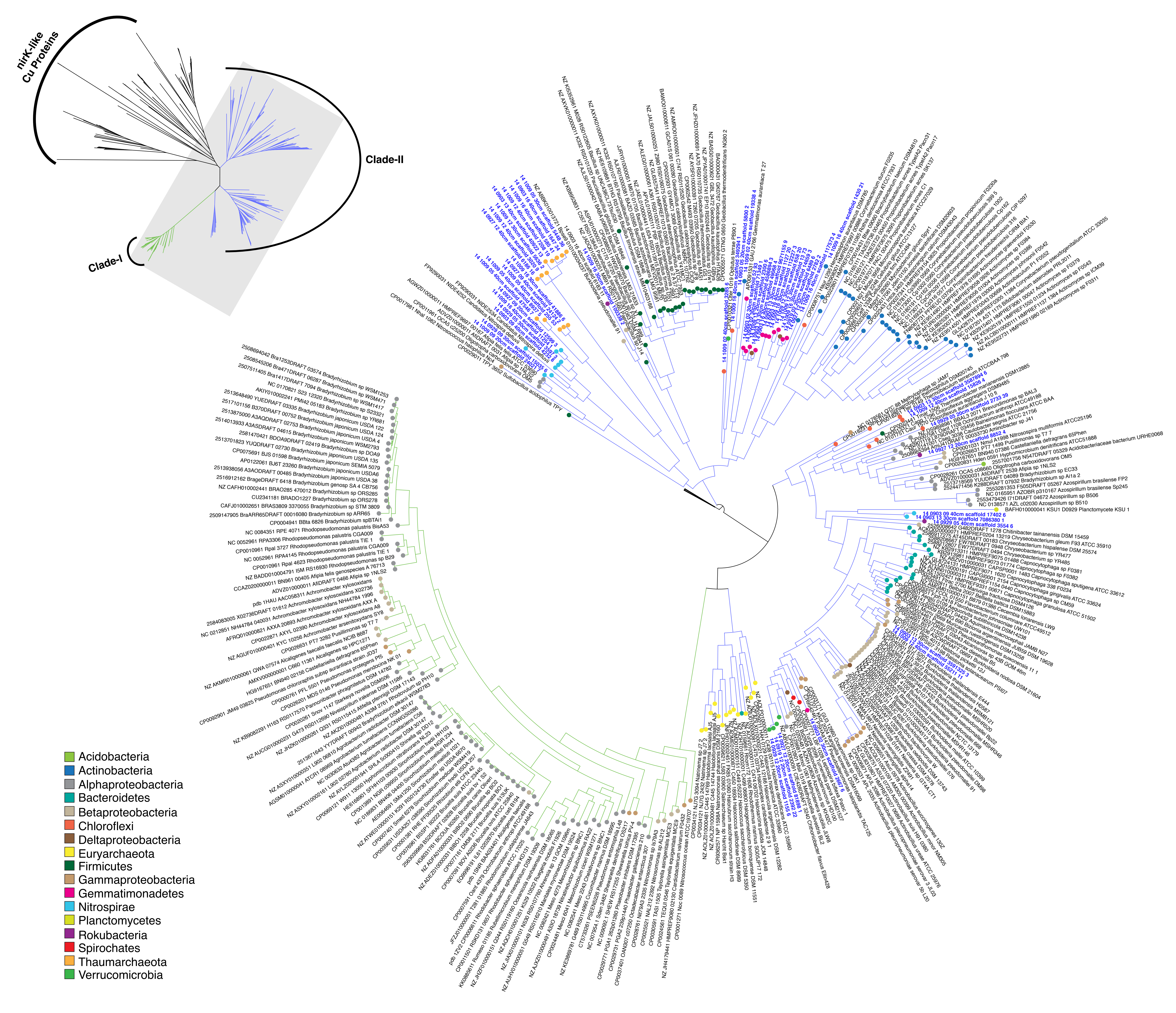
nirK-like
Cu Proteins
Clade-II
Clade-I
Acidobacteria
Actinobacteria
Alphaproteobacteria
Bacteroidetes
Betaproteobacteria
Chloroflexi
Deltaproteobacteria
Euryarchaeota
Firmicutes
Gammaproteobacteria
Gemmatimonadetes
Nitrospirae
Planctomycetes
Rokubacteria
Spirochates
Thaumarchaeota
Verrucomicrobia

Supplementary Figure 9 l Full nirK gene tree. (inset) The full unrooted tree for all nirK and nirK-like sequences identified by HMM search, and included references from Decleyre et al. The full tree was constructed from an alignment of 425 sequences. Colored clades and labels indicate membership in class I or II nirK sequence clades based on Decleyre et al. (methods). Grey box indicates the region displayed as a radial tree to the bottom right. **(radial tree)** Tree clade lines are colored identically to inset unrooted tree and represent nirK sequence class. Names in blue text indicate sequences identified in our study. Sequences from our study are named by their scaffold and gene number. Circles at tree nodes indicate phylum level membership of the organism encoding the nirK sequence (see key). For full newick tree see Supplementary Data 14.

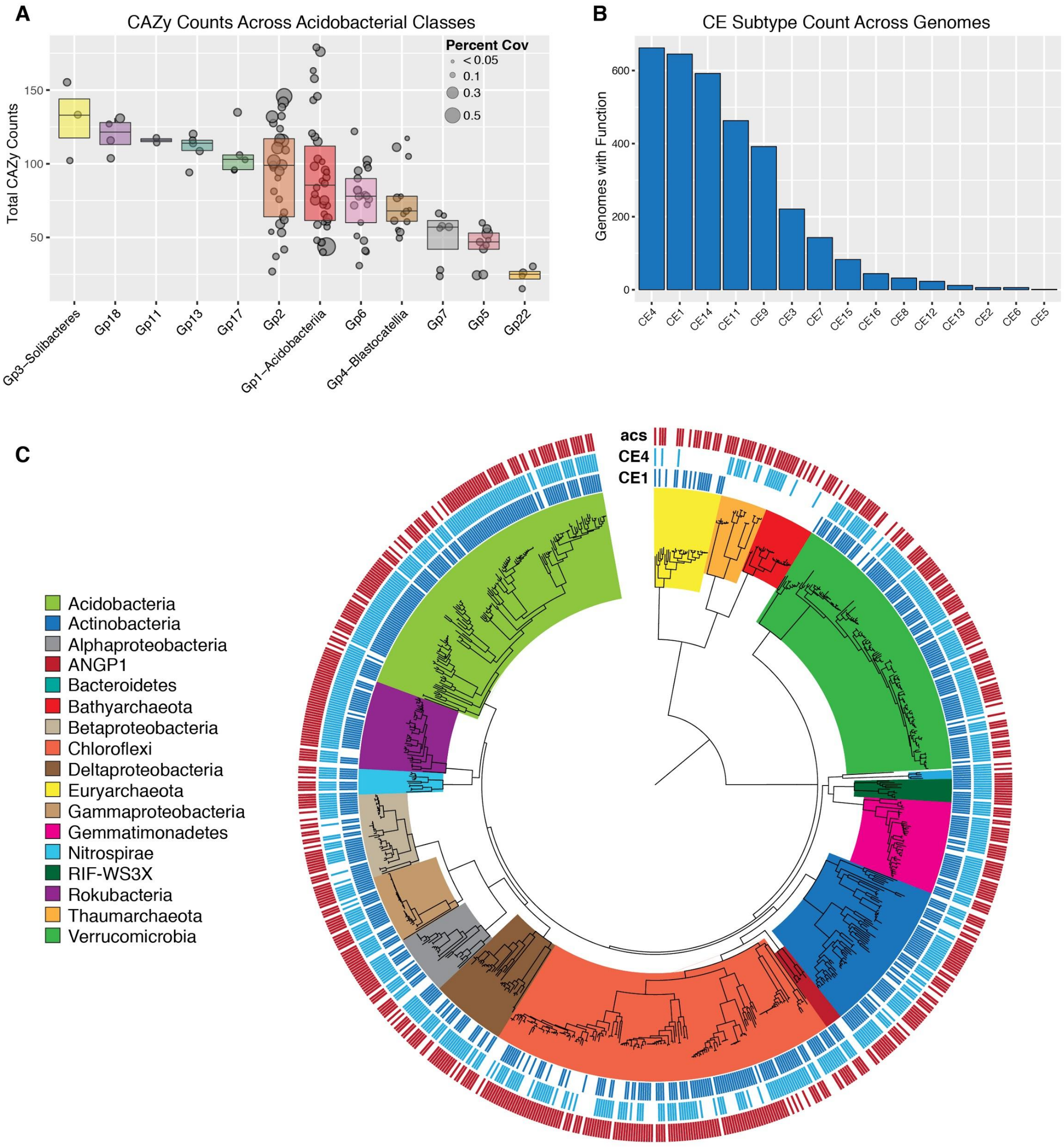

A
CAZy Counts Across Acidobacterial Classes
Total CAZy Counts
Percent Cov
< 0.05
0.1
0.3
0.5
50
100
150
Gp3–Solibacteres
Gp18
Gp11
Gp13
Gp17
Gp2
Gp1–Acidobacteriia
Gp6
Gp4–Blastocatellia
Gp7
Gp5
Gp22
B
CE Subtype Count Across Genomes
Genomes with Function
0
200
400
600
CE4
CE1
CE14
CE11
CE9
CE3
CE7
CE15
CE16
CE8
CE12
CE13
CE2
CE6
CE5
C
acs
CE4
CE1
Acidobacteria
Actinobacteria
Alphaproteobacteria
ANGP1
Bacteroidetes
Bathyarchaeota
Betaproteobacteria
Chloroflexi
Deltaproteobacteria
Euryarchaeota
Gammaproteobacteria
Gemmatimonadetes
Nitrospirae
RIF-WS3X
Rokubacteria
Thaumarchaeota
Verrucomicrobia

Supplementary Figure 10 l Supplementary CAZy Statistics. (A) Total counts of CAZy enzymes identified in the genomes of 12 acidobacterial classes reconstructed in this study (n = 138 genomes). Grey points indicate individual genomes and point size indicates relative percent of total coverage of a genome across all samples. Colors are arbitrary and differentiate acidobacterial classes. Boxes indicate median and 1st and 3rd quartile for a class. **(B)** Counts of genomes encoding at least one of the named carbohydrate esterase subtypes (n = 793 genomes analyzed). **(C)** Overlay of genomes that encode CE1, CE4, and acetyl-CoA synthetase (acs) onto the rp15 phylogenetic tree from Fig. 2. Ticks in the rings surrounding the tree indicate that the genome at that tree node contained a positive identification of the function indicated. Tree clades are colored by Phylum level taxonomy (see key). The tree was constructed using RaxML from a concatenated alignment of 15 co-located ribosomal proteins (L2, L3, L4, L5, L6, L14, L15, L16, L18, L24, S3, S8, S17, S19). The tree includes 852 genomes identified in our study where 8 or more ribosomal proteins were present.

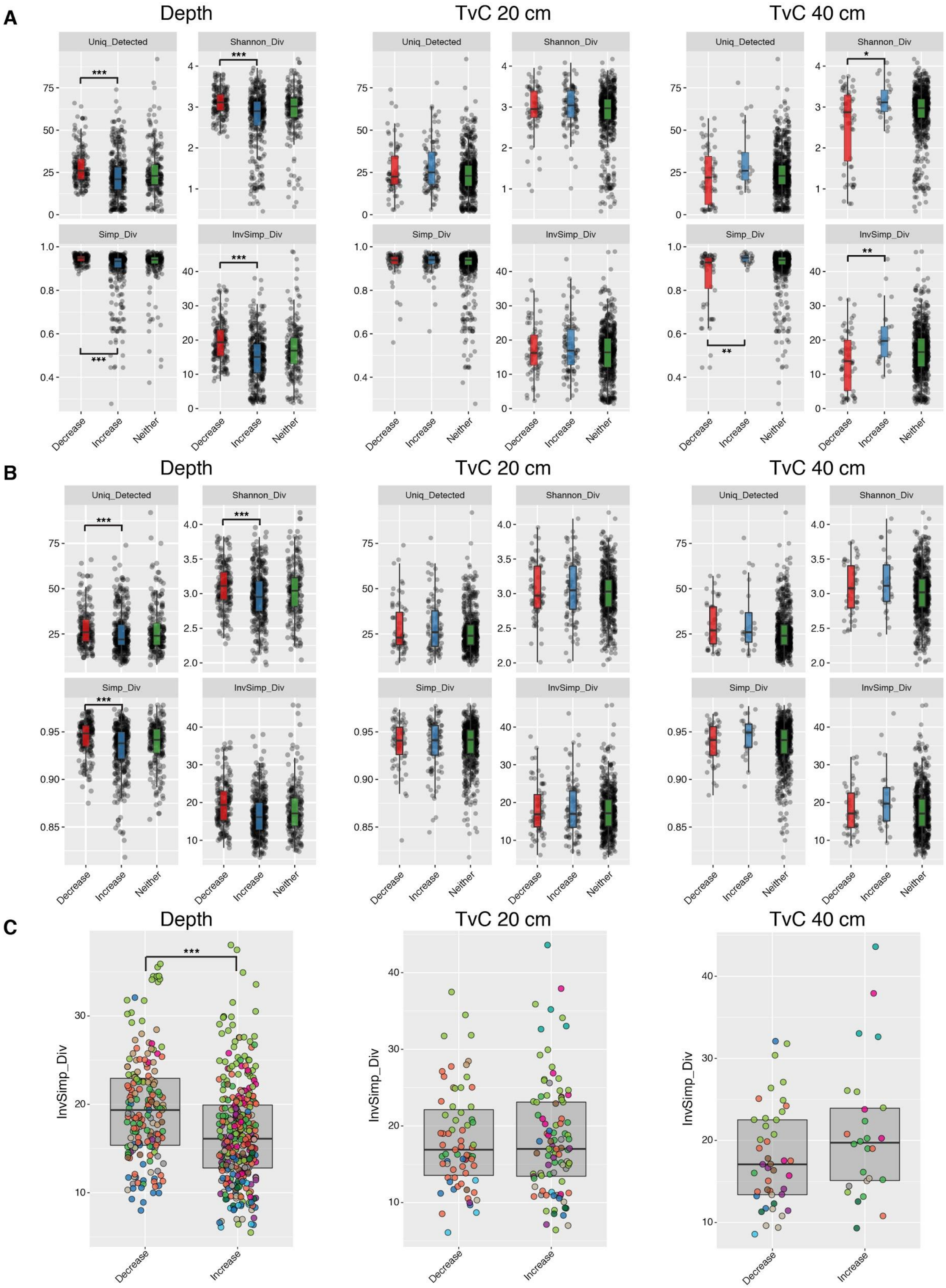

A
Depth
TvC 20 cm
TvC 40 cm
Uniq_Detected
Shannon_Div
Simp_Div
InvSimp_Div
Decrease
Increase
Neither
B
Depth
TvC 20 cm
TvC 40 cm
C
Depth
TvC 20 cm
TvC 40 cm
InvSimp_Div
Decrease
Increase

Supplementary Figure 11 | Supplementary Comparative CAZy Diversity Metrics. All calculated CAZy enzyme diversity metric distributions including: Uniq_Detected = Unique Enzymes per genome; Shannon_Div = Shannon Diversity; Simp_Div = Simpson Diversity; InvSimp_Div = Simpson diversity transformed to the inverse form (1/(1-Simpson Diversity)). Diversity metric distributions are displayed for genomes (points) that increase, decrease, or do not change (neither) in abundance with depth, extended rainfall treatment in 10-20 cm samples (TvC 20 cm), and extended rainfall treatment in 30-40 cm samples (TvC 40 cm). **(A)** All four Diversity metric distributions calculated across all 793 genomes analyzed in the study. Box colors are arbitrary and differentiate genome response groups. **(B)** All four Diversity metric distributions calculated across only the 722 bacterial genomes (Archaea removed) analyzed in the study. Box colors are arbitrary and differentiate genome response groups. **(C)** Zoomed in view of inverse Simpson diversity distributions calculated across only bacterial genomes (Archaea removed) increasing or decreasing in abundance with depth, in response to extended rainfall treatment in 10-20 cm samples (TvC 20 cm), and in response to extended rainfall treatment in 30-40 cm samples (TvC 40 cm). Points are colored by phylum (see Fig. 3). Across all figure panels sample numbers were: n_{depth} = 60 biologically independent samples, $n_{20cm\_treatment}$ = 24 biologically independent samples, $n_{40cm\_treatment}$ = 20 biologically independent samples. Across all figure panels the number of genomes analyzed were: n_{depth} = 570 independent genomes, $n_{20cm\_treatment}$ = 173 independent genomes, $n_{40cm\_treatment}$ = 85 independent genomes. For all plots boxes indicate median and 1st and 3rd quartile for points. Box whiskers, in panels A and B, encompass 1.5*interquartile range. A black star between box plots indicates a statistically difference, and all statistics were adjusted for multiple testing using false discovery rate (two-sided Wilcoxon test; * FDR ≤ 0.05, ** FDR ≤ 0.01, *** FDR ≤ 0.001; For exact FDR values see Supplementary Table 16).

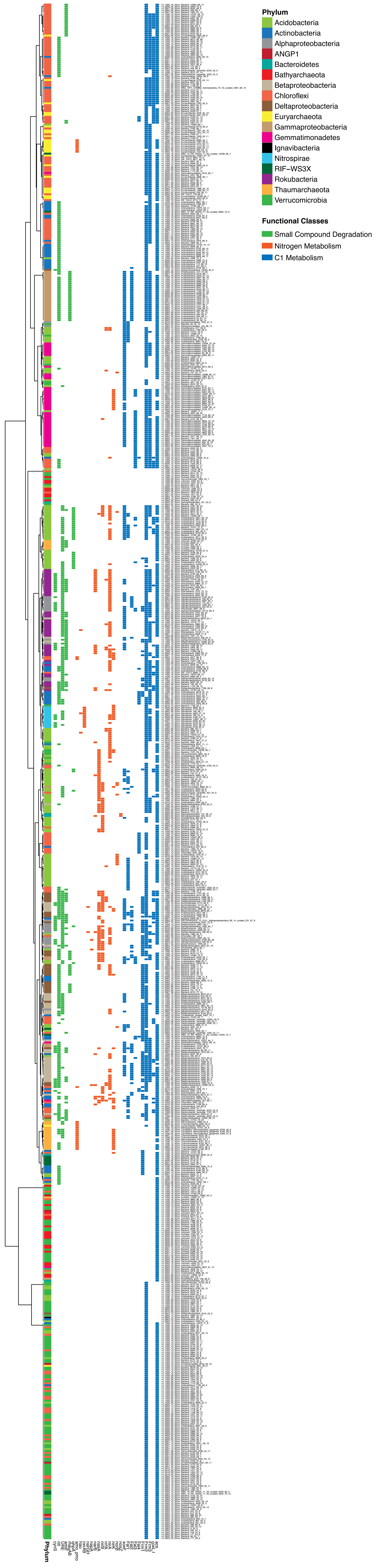
Phylum
Acidobacteria
Actinobacteria
Alphaproteobacteria
ANGP1
Bacteroidetes
Bathyarchaeota
Betaproteobacteria
Chloroflexi
Deltaproteobacteria
Euryarchaeota
Gammaproteobacteria
Gemmatimonadetes
Ignavibacteria
Nitrospirae
RIF−WS3X
Rokubacteria
Thaumarchaeota
Verrucomicrobia
Functional Classes
Small Compound Degradation
Nitrogen Metabolism
C1 Metabolism
Phylum

Supplementary Figure 12 l Co-Occurrence of Targeted Carbon and Nitrogen Transformation Functions. The occurrence of all 29 targeted carbon and nitrogen transformation functions in all 793 genomes analyzed for metabolic traits in our study (See Supplementary Tables 9 and 10). Genomes are clustered based on presence/absence of all 29 functions using binary distance and Ward hierarchical grouping. Each row represents one genome with genome bin names present at the end of the row. Phylum-level taxonomy is indicated by a colored bar at the beginning of a row. Individual functional traits are noted at the bottom of the plot and are colored arbitrarily based on general functional class.

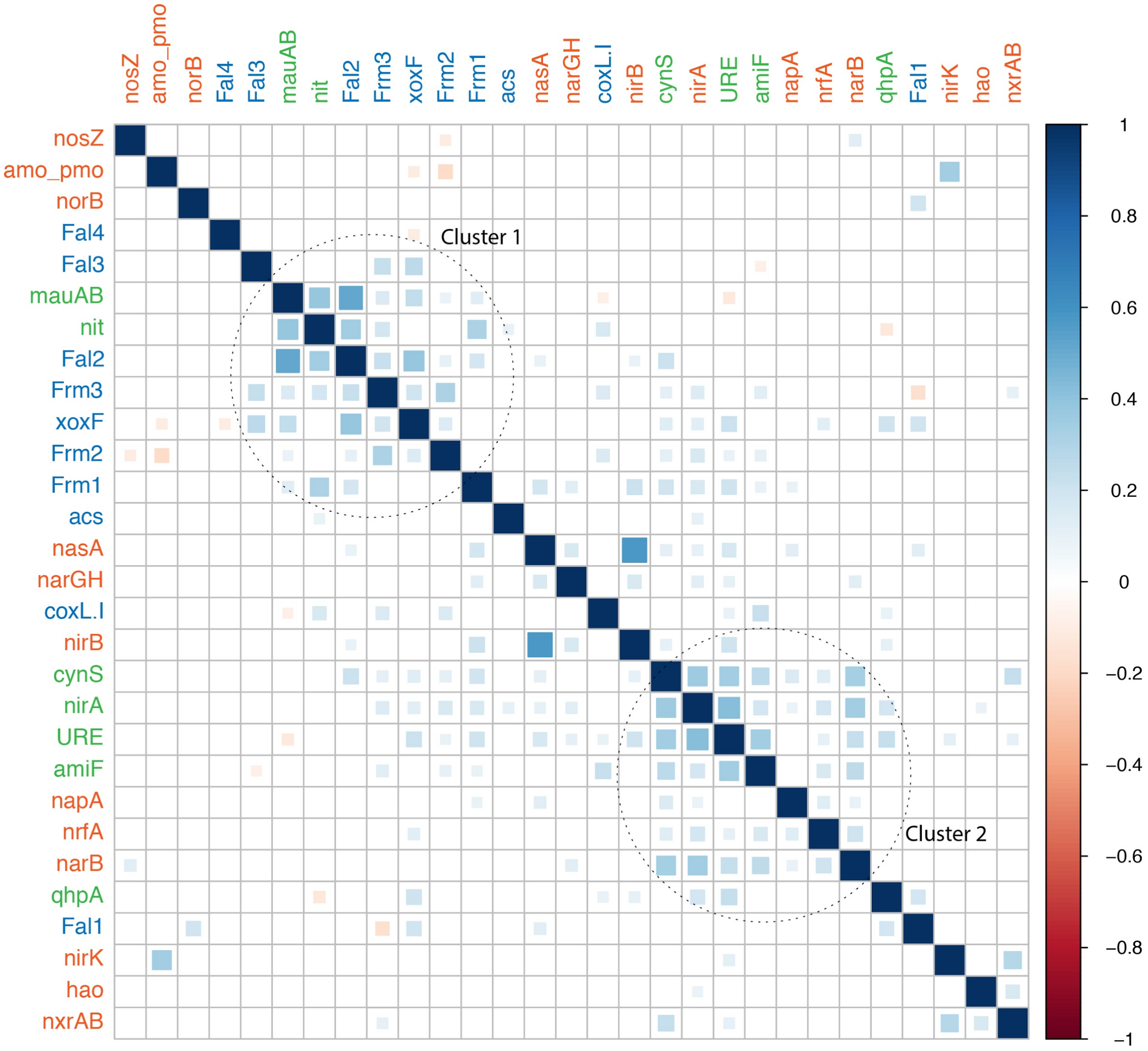

nosZ
amo_pmo
norB
Fal4
Fal3
mauAB
nit
Fal2
Frm3
xoxF
Frm2
Frm1
acs
nasA
narGH
coxL.I
nirB
cynS
nirA
URE
amiF
napA
nrfA
narB
qhpA
Fal1
nirK
hao
nxrAB
Cluster 1
Cluster 2
1
0.8
0.6
0.4
0.2
0
−0.2
−0.4
−0.6
−0.8
−1

Supplementary Figure 13 l Co-occurrence Correlation of Targeted Carbon and Nitrogen Transformation Functions. Significant spearman rank correlations between all 29 targeted carbon and nitrogen transformation functions in genomes analyzed for metabolic traits (n = 793 independent genomes) in our study (See Supplementary Tables 9 and 10). Both upper and lower identical sides of the correlation triangle are shown for ease of viewing. A square in the grid indicates a significant correlation, and correlation p-values were corrected for multiple testing using false discovery rate (two-sided rank correlation t-test; FDR ≤ 0.05 cutoff for inclusion in figure). Square size and color intensity reflect the magnitude of the correlation as noted in the color legend. Blue squares indicate positive correlations and red squares indicate negative correlation. Metabolic functions are colored based on general functional class as in Supplementary Fig. 12 (Green = small compound degradation; Orange = nitrogen metabolism; Blue = C1 metabolism). Human defined clusters are bounded by circles and cluster number is indicated proximally.

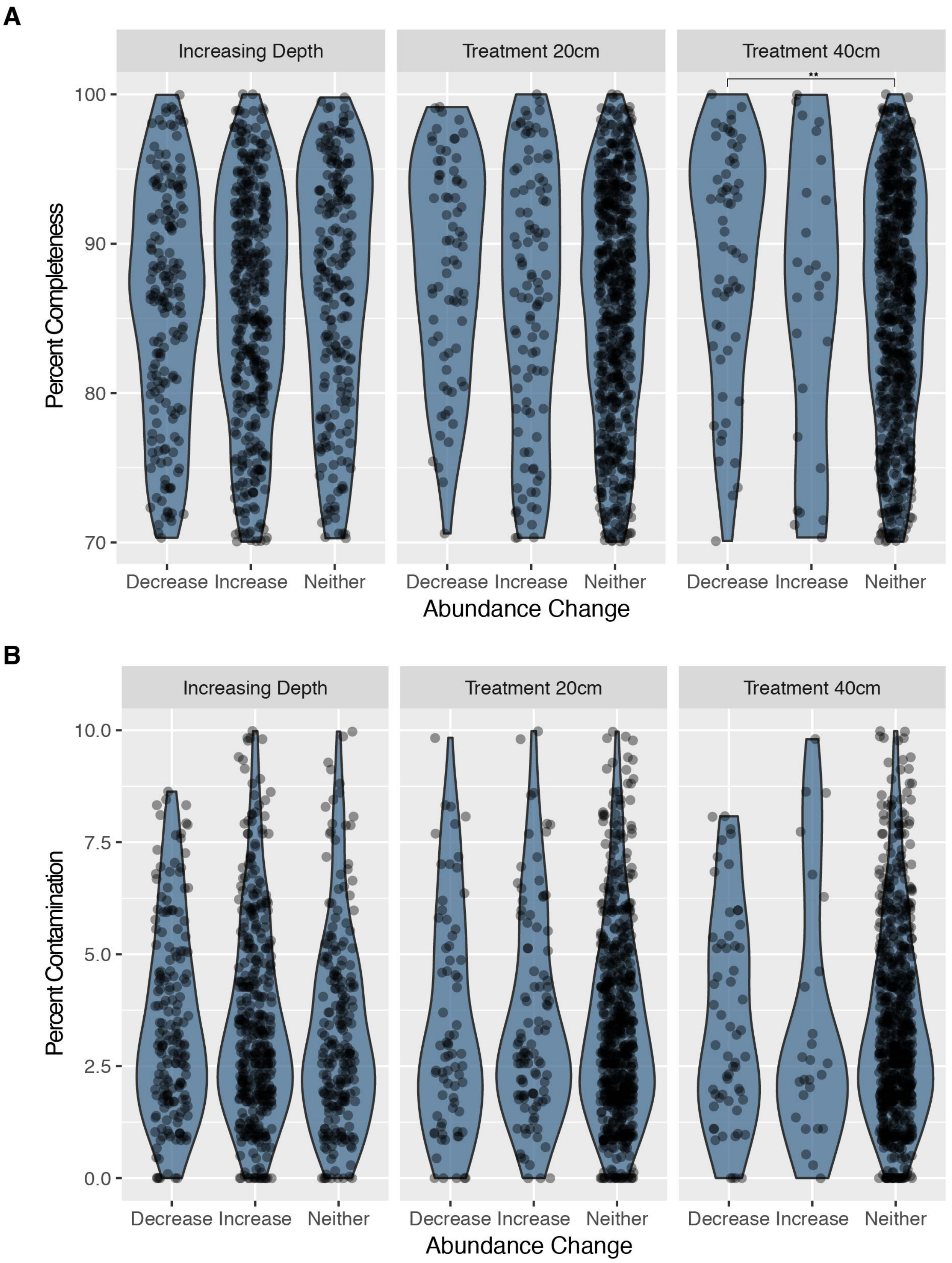
A
Increasing Depth
Treatment 20cm
Treatment 40cm
**
Percent Completeness
100
90
80
70
Decrease Increase Neither
Decrease Increase Neither
Decrease Increase Neither
Abundance Change
B
Increasing Depth
Treatment 20cm
Treatment 40cm
Percent Contamination
10.0
7.5
5.0
2.5
0.0
Decrease Increase Neither
Decrease Increase Neither
Decrease Increase Neither
Abundance Change

Supplementary Figure 14 I Analysis of Equivalence for all Compared Genome Sets. The distributions of estimated completeness and contamination for the three genome response groups (Decrease = decreasing in abundance; Increase = increasing in abundance, Neither = no change in abundance) compared across each tested condition (Increasing Depth; Treatment 20cm = extended rainfall treatment at 10-20 cm depth; Treatment 40cm = extended rainfall treatment at 30-40 cm depth), for functional enrichment analysis. Across all figure panels sample numbers were: n_{depth} = 60 biologically independent samples, $n_{20cm\_treatment}$ = 24 biologically independent samples, $n_{40cm\_treatment}$ = 20 biologically independent samples. Across all figure panels the number of genomes analyzed were: n_{depth} = 570 independent genomes, $n_{20cm\_treatment}$ = 173 independent genomes, $n_{40cm\_treatment}$ = 85 independent genomes. Violin plots (blue) indicate distribution shape with larger thickness indicating higher density of genomes. Points show values for individual genomes. **(A)** Distributions of estimated genome completeness. Brackets above plots indicate a statistically significant difference, and all p-values were corrected for multiple testing with false discovery rate (FDR) (two-sided Wilcoxon rank sum test; ** FDR ≤ 0.01). **(B)** Distributions of estimated genome completeness. Brackets above plots indicate a statistically significant difference, and all p-values were corrected for multiple testing with FDR (two-sided Wilcoxon rank sum test; no significant differences detected). For all summary statistics, and exact p and FDR values for comparisons see Supplementary Table 19.

Supplementary Table Legends

[See Supplementary Table Excel file in manuscript supplement]

Supplementary Table 1 I Sample Metadata and Assembly Statistics. Tabular list of metadata and assembly statistics for all metagenomics samples analyzed in the study (n = 60 biologically independent samples). Table lines 60-74 show summary statistics and totals for assemblies across all samples.

Supplementary Table 2 I All Identified rpS3 SGs, Predicted Phylogeny, and Abundance Data. Tabular list of all rpS3 based species groups (SGs; n = 3325 independent SGs) identified across all metagenomics samples (n = 60 independent samples). List includes the names of the rpS3 sequences identified as the 99% ID centroid for each SG and the longest rpS3 containing scaffold present in each SG, which was used for abundance mapping and quantification. For each SG, inferred phylogenetic assignment, total coverage, and relative fractional coverage are noted. Exact statistical significance values from the DEseq tests are corrected for multiple testing using false discovery rate (FDR) and noted if FDR ≤ 0.05 (n_{depth} = 60 biologically independent samples, two-sided likelihood ratio test followed by two-sided linear model slope significance; $n_{20cm\_treatment}$ = 24 biologically independent samples and $n_{40cm\_treatment}$ = 20 biologically independent samples, two-sided Wald test). If an SG is associated with a genomic bin the bin name is also noted.

Supplementary Table 3 I Raw Counts of Reads from Each Sample Mapped to the Longest Contig of an SG Cluster. Raw read counts per contig derived from mapping all reads in each metagenomic sample separately against the longest rpS3 containing scaffold present in each

SG (n = 3325 independent scaffolds). Scaffold names and their associated SG identifier are in rows and read counts derived from each individual sample are in columns.

Supplementary Table 4 l SG Coverage of Reads from Each Sample Normalized to Total Sequencing Depth per Sample. Normalized per base pair read coverage for all longest rpS3 containing scaffolds present in each SG (n = 3325 independent scaffolds). Normalized coverage for each SG in each sample (n = 60 independent samples) was derived using the following formula: (per base pair coverage of SG in sample / reads sequenced in sample) x 100,000,000. Contig names and their associated SG identifier are in rows and the normalized coverage derived for each SG from each individual sample are in columns.

Supplementary Table 5 l All Non-Redundant Bins Identified in the Study and Associated Information. Tabular list of all non-redundant genome bins, containing an SG sequence, identified in the study (n = 896 independent genomes). If a bin was included in our metabolic analysis is noted (Metabolism Analysis = TRUE). Metabolism was only analyzed in bins with estimated completeness ≥ 70% and estimated contamination ≤ 10% (n = 793 independent bins). Inferred phylogenetic assignment for bins is noted, as well as the method used to derive assignment (see methods). The total coverage, and relative fractional coverage calculated for the SG associated with a bin are also noted. Exact statistical significance values from the DEseq tests are repeated from Supplementary Table 2 for ease of access. These values are corrected for multiple testing using false discovery rate (FDR) and noted if FDR ≤ 0.05 (n_{depth} = 60 biologically independent samples, two-sided likelihood ratio test followed by two-sided linear model slope significance; $n_{20cm\_treatment}$ = 24 biologically independent samples and $n_{40cm\_treatment}$ = 20 biologically independent samples, two-sided Wald test). Genome size, GC, scaffold count, and estimated completeness and contamination statistics are also included.

Supplementary Table 6 I All 16S Genes Identified in Bins and Associated Taxonomy. Tabular list of 16S sequences identified in non-redundant genome bins (n = 896 independent genomes) across all samples in our study (n = 60 independent samples). Table indicates the gene name for each sequence, associated bin name, and proposed taxonomy from search against the SILVA 16S sequence database.

Supplementary Table 7 I Proteomics Summary Data and Sample Metadata. Tabular list of metadata and aggregate spectral count data for all metaproteomics samples analyzed in the study (n = 20 biologically independent samples). The table includes total proteins identified in each sample, total spectral counts per sample, the maximum spectral counts assigned to a single protein in a sample, and the average number of spectral counts across all proteins in a sample. Table lines 24-28 show summary statistics and totals for spectral count data.

Supplementary Table 8 I Abundance Ranked Proteomics Orthology Groups Found in ≥ 5 Samples. Tabular list of all protein functional orthology groups (see methods) identified in ≥ 5 proteomic samples (n = 20 biologically independent samples) ranked by mean spectral count. Under “Final Annotation” K numbers indicate KEGG database orthology identifiers. Exact statistical significance values for the test assessing functional over-enrichment in our dataset vs. the KEGG database are noted and corrected for multiple testing using false discovery rate (FDR) (n = 377 independent functional orthology groups, one-sided hypergeometric enrichment test). For full proteomics count information see Supplementary Data 9.

Supplementary Table 9 I Gene Search Methods and Identification Criteria for Specifically Targeted C1 and Nitrogen Metabolic Pathways. Tabular list showing the marker genes, and

detection method used, to identify the presence of the 29 targeted C1 and nitrogen metabolic functions analyzed in the study (Also see methods).

Supplementary Table 10 l Presence of Targeted C1 and Nitrogen Metabolic Functions in 793 Genomes Passing Thresholds for Metabolic Profiling. Table indicating the binary presence or absence of the 29 targeted C1 and nitrogen metabolic functions analyzed in the study across all genomes subjected to metabolic analysis (n = 793 independent genomes). Presence in genome is indicated by a 1 and absence is indicated by a 0. Phylum assignment, and responses to depth and treatment are noted for each bin for ease of data association.

Supplementary Table 11 l CAZy Classes Identified in All 793 Genomes Passing Thresholds for Metabolic Profiling. Table indicating the count of each of 246 CAZy functions identified in the study across all genomes subjected to metabolic analysis (n = 793 independent genomes). Each count indicates a unique gene locus. Phylum assignment, and responses to depth and treatment are noted for each bin for ease of data association. For raw CAZy annotation output see Supplementary Data 11.

Supplementary Table 12 l KEGG KO Assignments for All 793 Genomes Passing Thresholds for Metabolic Profiling. Table indicating the count of each of 5,435 KEGG functions identified in the study across all genomes subjected to metabolic analysis (n = 793 independent genomes). Each count indicates a unique gene locus. Phylum assignment, and responses to depth and treatment are noted for each bin for ease of data association. For raw KEGG annotation output see Supplementary Data 10.

Supplementary Table 13 I Functions Identified Using Phylogenetic Placement. Functions where phylogenetic reconstruction was used to assign the enzyme functional subtype (see methods). Functions included in this table are methanol dehydrogenase (xoxF), carbon monoxide dehydrogenase (coxL), and dissimilatory nitrite reductase (nirK). Each individual identified instance of a function across all genomes subjected to metabolic analysis (n = 793 independent genomes) are listed in rows. Locus IDs, associated genome bin IDs, HMM cutoffs used to initially identify the functional class, and individual HMM scores for each protein are also noted.

Supplementary Table 14 I Enrichment of Phyla Across Depth and Treatment. Full results for the counts and enrichment of phylum level groups for each of the three response groups (Increase, Decrease, and Neither) across the three conditions tested (Depth, Treatment - 20cm, and Treatment - 40 cm). For each phylum, the number of genomes Decreasing, Increasing, or not responding (Neither) are noted for each condition, as well as the total number of genomes in each of those categories and the total number of genomes with that phylum level assignment. Phyla that did not have a member increasing or decreasing under a condition were dropped from the analysis. Fractional difference was calculated as the absolute value of: Decrease / Total Decrease) - (Increase / Total Increase). Log2 odds ratios, exact p-values, and p-values corrected for multiple testing using false discovery rate (FDR) are reported for fisher testing between the Decrease, Increase, and Neither counts for each phylum (two-sided Fisher's Exact Test, n = 793 independent genomes). Phyla with a Fisher FDR ≤ 0.1 were subjected to custom permutation testing (see methods), and exact p-values and p-values corrected for multiple testing with FDR are reported (two-sided permutation enrichment test, n = 793 independent genomes; see methods). Phyla where permutation test FDR values were ≤ 0.05 are considered significant and colored in red text.

Supplementary Table 15 I Enrichment of Genomes with Targeted Functions Across Depth and Treatment. Full results for the counts and enrichment of targeted metabolic functions (n = 29 independent functions) for each of the three response groups (Increase, Decrease, and Neither) across the three conditions tested (Depth, Treatment - 20cm, and Treatment - 40 cm). For each function, the number of genomes Decreasing, Increasing, or not responding (Neither) are noted for each condition, as well as the total number of genomes in each of those categories and the total number of genomes carrying the function. Functions that did not occur in genomes increasing or decreasing under a condition were dropped from the analysis. Fractional difference was calculated as the absolute value of: Decrease / Total Decrease) - (Increase / Total Increase). Log2 odds ratios, exact p-values, and p-values corrected for multiple testing using false discovery rate (FDR) are reported for fisher testing between the Decrease, Increase, and Neither counts for each function (two-sided Fisher's Exact Test, n = 793 independent genomes). Functions with a Fisher FDR ≤ 0.1 were subjected to custom permutation testing (see methods), and exact p-values and p-values corrected for multiple testing with FDR are reported (two-sided permutation enrichment test, n = 793 independent genomes; see methods). Functions where permutation test FDR values were ≤ 0.05 are considered significant and colored in red text.

Supplementary Table 16 I Diversity Analysis of CAZy Enzymes Across Differentially Abundant Genomes in Depth and Treatment. Full results for the analysis of bulk CAZy enzyme diversity (n = 246 independent CAZy functions) for each of the three response groups (Increase, Decrease, and Neither) across the three conditions tested (Depth, Treatment - 20cm, and Treatment - 40 cm). Analyses are presented for the full set of genomes metabolically analyzed in our study (n = 793 independent genomes) and this same set with archaeal

genomes removed (n = 722 independent bacterial genomes). For each analysis, the mean and standard deviation for the 4 diversity metrics are presented for each of the three response groups (Depth: $n_{Decrease}$ = 179 independent genomes, $n_{Increase}$ = 391 independent genomes, $n_{Neither}$ = 223 independent genomes; Treatment - 20 cm: $n_{Decrease}$ = 72 independent genomes, $n_{Increase}$ = 101 independent genomes, $n_{Neither}$ = 620 independent genomes; Treatment - 40 cm: $n_{Decrease}$ = 59 independent genomes, $n_{Increase}$ = 26 independent genomes, $n_{Neither}$ = 708 independent genomes). All response groups were first compared with the Kruskal-Wallis (KW) test and the exact KW p-values and their corrected equivalents using false discovery rate (FDR) are noted (two-sided Kruskal-Wallis test). For instances where KW FDR ≤ 0.1 a Wilcoxon rank sum test was conducted only between groups of genomes that Increase or Decrease (two-sided Wilcoxon test). Exact significance values for the Wilcoxon test, corrected for multiple testing using FDR, are noted. Wilcoxon FDR values ≤ 0.05 were considered significant and are presented in red text.

Supplementary Table 17 I Enrichment of CAZy Enzymes Across Differentially Abundant Genomes in Depth and Treatment. Full results for individual CAZy enzyme class enrichments (n = 246 independent CAZy functions) for each of the three response groups (Increase, Decrease, and Neither) across the three conditions tested (Depth, Treatment - 20cm, and Treatment - 40 cm). The mean enzyme counts for genomes in each response group are given (Depth: $n_{Decrease}$ = 179 independent genomes, $n_{Increase}$ = 391 independent genomes, $n_{Neither}$ = 223 independent genomes; Treatment - 20 cm: $n_{Decrease}$ = 72 independent genomes, $n_{Increase}$ = 101 independent genomes, $n_{Neither}$ = 620 independent genomes; Treatment - 40 cm: $n_{Decrease}$ = 59 independent genomes, $n_{Increase}$ = 26 independent genomes, $n_{Neither}$ = 708 independent genomes). The total counts of a CAZy class across all genomes analyzed in our study is also noted (n = 793 independent genomes). Log2 odds ratios were calculated between the number

of organisms that Decreased/Increased vs. the background of their respective sets. Exact p-values and their equivalents corrected for multiple testing with false discovery rate (FDR) are presented for the first comparison of a CAZy category across all response groups (two-sided Kruskal-Wallis test). For CAZy classes where KW FDR ≤ 0.1 a Wilcoxon rank sum test was conducted only between groups of genomes that Increase or Decrease (two-sided Wilcoxon test). Exact significance values for the Wilcoxon test, corrected for multiple testing using FDR, are noted. Wilcoxon FDR values ≤ 0.05 were considered significant and are presented in red text.

Supplementary Table 18 I KEGG KO Groups Identified as Discriminatory and Differentially Enriched Between Genomes that Change in Abundance with Increasing Depth. Summary of random forest based feature selection (Boruta; see methods) for identifying KEGG functions that had a significant association with genomes either increased or decreased in abundance with depth. For each KEGG functional orthology group the number of genomes with the function in the increasing or decreasing depth response group is noted. Log2 Odds were calculated between the number of organisms that Decreased/Increased vs the background of their respective sets. Exact p-values for the enrichment of a KEGG orthology group and their equivalents corrected for multiple testing using false discovery rate (FDR) are presented (two-sided Wilcoxon test).

Supplementary Table 19 I Completeness and Contamination Summary Statistics and Comparisons for Genome Groups Compared in all Analyses. Summary statistics for estimated genome completeness and contamination across all genomes that were metabolically analyzed in our study (n = 793 independent genomes). Mean completeness, contamination, and the number of genomes analyzed are displayed for each condition (Depth, Treatment - 20cm,

and Treatment - 40 cm) and response group (Increase, Decrease, and Neither). Test statistics, exact p-values, and their equivalents corrected for multiple testing using false discovery rate (FDR) are shown for genome completeness and contamination comparisons across the three response groups (two-sided Kurskal-Wallis test). In instances where the Kurskal-Wallis FDR ≤ 0.05, post-hoc testing was carried out between specific response groups (two-sided pairwise Wilcoxon test). The absolute mean difference was calculated by taking the absolute value of the difference in the mean completeness of the groups in the comparisons indicated. Significant results are noted in red text.

Supplementary Dataset Legends

[See Supplementary Datasets in manuscript supplement]

Supplementary Dataset 1 l All rpS3 Centroid Sequences. This dataset contains all 3,325 rpS3 centroid protein sequences used as the representatives for each Species Group (SG) cluster in FASTA format (See Supplementary Table 2 and methods).

Supplementary Dataset 2 l All rpS3 Containing Longest Scaffolds for Species Groups. This dataset contains all 3,325 longest rpS3 containing DNA scaffolds for each Species Group (SG) cluster in FASTA format (See Supplementary Table 2 and methods). These contigs were used as the mapping targets to determine the relative abundance of all SGs in the study.

Supplementary Dataset 3 l Full rpS3 Protein Tree with Reference Sequence. This dataset is a newick format tree file for the full rpS3 protein tree. The rpS3 sequences from our study used in this tree are those from Supplementary Dataset 1. The full tree was constructed using FastTree from an alignment of 5,649 rpS3 protein sequences (3,325 identified in our data and 2,324 reference sequences). This tree was used to produce rough phylogenetic assignments. FastTree support values are included (1,000 FastTree bootstrap replicates).

Supplementary Dataset 4 l rpS3 Protein Tree with Only Sequences from Our Study. This dataset is a newick format tree file for a maximum likelihood reconstruction containing only the 3,325 rpS3 sequences identified in our study. The rpS3 sequences from our study used in this tree are those from Supplementary Dataset 1. The tree was constructed using RaxML from an alignment of 3,325 sequences, and was used to generate phylogenetic weights for the weighted

Unifrac distance metric in the paper (see Figure 1 and methods). Bootstrap support values are included (124 bootstrap replicates).

Supplementary Dataset 5 I Full Concatenated Ribosomal Protein Tree with References. This dataset is a newick format tree file for the full maximum likelihood reconstruction of the 15 co-located ribosomal proteins (L2, L3, L4, L5, L6, L14, L15, L16, L18, L24, S3, S8, S17, S19) used for genome phylogenetic assignment. The tree includes 1,916 genomes where 8 or more ribosomal proteins were identified (852 genomes identified in our study and 1,064 references). The tree was constructed with RAxML and bootstrap values are included in the file (142 bootstrap replicates). Genome names from our study in this tree can be found in Supplementary Table 5.

Supplementary Dataset 6 I Full Concatenated Ribosomal Protein Alignment for Ribosomal Protein Tree. This dataset contains a FASTA protein file with the concatenated set of 15 co-located ribosomal proteins (L2, L3, L4, L5, L6, L14, L15, L16, L18, L24, S3, S8, S17, S19) used in construction of the tree in Supplementary Data 5. The file includes concatenated ribosomal protein alignments from 1,916 genomes where 8 or more ribosomal proteins were identified (852 genomes identified in our study and 1,064 references). Alignments were constructed for each protein individually with muscle, and alignments were stripped of columns with 95% gap characters and concatenated using Geneious (see methods).

Supplementary Dataset 7 I Individual Ribosomal Protein Sequence Sets. This dataset is a zip archive that contains separate protein files for each of the 15 co-located ribosomal proteins (L2, L3, L4, L5, L6, L14, L15, L16, L18, L24, S3, S8, S17, S19) used for tree construction in our

study. The proteins come from 1,916 genomes where 8 or more ribosomal proteins were identified (852 genomes identified in our study and 1,064 references).

Supplementary Dataset 8 l All 16S Sequences Identified in Our Study. This dataset is a FASTA DNA file containing all 296 16S sequences identified in non-redundant genome bins (n = 896 independent genomes) across all samples in our study (n = 60 independent samples). Associations of sequence names with their SILVA taxonomy, final inferred phylogenetic assignment, and genome bin can be found in Supplementary table 6.

Supplementary Dataset 9 l Full Proteomics Protein Count Data. This dataset contains the spectral counts, associated metadata, and associated functional annotations for all 55,665 proteins in tabular format detected via metaproteomics (n = 20 independent samples) in our study (See methods and Supplementary Tables 7-8).

Supplementary Dataset 10 l All KEGG HMM Based Annotations for Metabolically Analyzed Genomes. This dataset contains all hits in tabular format for KEGG sequence derived hidden markov models (HMMs; see methods) searched against all metabolically analyzed genomes in our study (n = 793 independent genomes; Also, see Supplementary Tables 5, 9, 10, and 12). Included in the table for each protein are the origin genome bin, HMM e-values, HMM scores, HMM cutoff scores, and KEGG Orthology (KO) assignment for each protein if a hit was found. In total the file includes results for 3,297,702 analyzed proteins.

Supplementary Dataset 11 l All Filtered dbCAN HMM hits for Metabolically Analyzed Genomes. This dataset contains filtered hits in tabular format for dbCAN carbohydrate active enzyme (CAZy) HMM models searched against all metabolically analyzed genomes in our study

(n = 793 independent genomes; Also, see Supplementary Tables 5 and 11). Hits were filtered to an e-value ≤ $1e^{-14}$, HMM coverage ≥ 0.3, and only a single instance of a CAZy class was reported for each protein (see methods). Included in the table for each protein are the origin genome bin, HMM e-values, HMM alignment information, and CAZy class assignments. In total the file includes results for 38,139 unique proteins.

Supplementary Dataset 12 I Full pqq-Alcohol Dehydrogenase Protein Tree. This dataset contains the full newick format tree constructed from an alignment of all pqq-containing alcohol dehydrogenase protein sequences identified by HMM search in our study (see methods), and reference sequences from Keltjens et al. and Taubert et al. The full tree contains 2,218 sequences. Sequences from our study are named by their scaffold and gene number, and reference sequences from Keltjens et al. and Taubert et al. are named by NCBI accession numbers. For reference sequences ADH, XoxF, and MxaF subtypes are indicated in the sequence name. The tree was constructed using FastTree and FastTree support values are included (1,000 FastTree bootstrap replicates). For associated information also see Supplementary Tables 10, 13, and Supplementary Figure 7.

Supplementary Dataset 13 I Full CoxL Carbon Monoxide Dehydrogenase-like Protein Tree. This dataset contains the full nexus format tree constructed from an alignment of all coxL-like protein sequences identified by HMM search in our study (see methods), and reference sequences from Quiza et al. The full tree contains 1,944 sequences. Sequences from our study are named by their scaffold and gene number, and reference sequences from Quiza et al. are named by NCBI accession numbers. The tree was constructed using FastTree and FastTree support values are included (1,000 FastTree bootstrap replicates). For associated information also see Supplementary Tables 10, 13, and Supplementary Figure 8.

Supplementary Dataset 14 l Full NirK Nitrite Reductase Protein Tree. This dataset contains the full newick format tree constructed from an alignment of all nirK and nirK-like protein sequences identified by HMM search in our study (see methods), and reference sequences from Decleyre et al. The full tree contains 425 sequences. Sequences from our study are named by their scaffold and gene number, and sequences from Decleyre et al. are named by NCBI accession numbers. The tree was constructed using FastTree and FastTree support values are included (1,000 FastTree bootstrap replicates). For associated information also see Supplementary Tables 10, 13, and Supplementary Figure 9.

Supplementary References

51. Hug, L. A. *et al.* A new view of the tree of life. *Nature Microbiology* 1–6 (2016). doi:10.1038/nmicrobiol.2016.48

52. Edgar, R. C. MUSCLE: multiple sequence alignment with high accuracy and high throughput. *Nucleic Acids Res.* **32,** 1792–1797 (2004).

53. Eddy, S. R. Accelerated Profile HMM Searches. *PLoS Comput Biol* **7,** e1002195–16 (2011).

54. Lomolino, M. V. Ecology's most general, yet protean 1 pattern: the species-area relationship. *Journal of Biogeography* **27,** 17–26 (2000).

55. Oksanen, J., Blanchet, F. G., Kindt, R. & Legendre, P. *R Package 'vegan': Community Ecology Package. R Package version 2.2–0*. (2014).

56. Chiu, C.-H., Wang, Y.-T., Walther, B. A. & Chao, A. An improved nonparametric lower bound of species richness via a modified good-turing frequency formula. *Biom* **70,** 671–682 (2014).

57. Price, M. N., Dehal, P. S. & Arkin, A. P. FastTree 2--approximately maximum-likelihood trees for large alignments. *PLoS ONE* **5,** e9490 (2010).

58. McMurdie, P. J. & Holmes, S. phyloseq: An R Package for Reproducible Interactive Analysis and Graphics of Microbiome Census Data. *PLoS ONE* **8,** e61217–11 (2013).

59. Nguyen, L.-T., Schmidt, H. A., Haeseler, von, A. & Minh, B. Q. IQ-TREE: A Fast and Effective Stochastic Algorithm for Estimating Maximum-Likelihood Phylogenies. *Mol. Biol. Evol.* **32,** 268–274 (2014).

60. Wickham, H. *ggplot2: Elegant Graphics for Data Analysis*. (Springer Science & Business Media, 2009). doi:10.1007/978-0-387-98141-3

61. Love, M. I., Huber, W. & Anders, S. Moderated estimation of fold change and dispersion

for RNA-seq data with DESeq2. *Genome Biol* **15,** 550 (2014).

62. Benjamini, Y. & Hochberg, Y. Controlling the false discovery rate: a practical and powerful approach to multiple testing. *Journal of the Royal Statistical Society Series B …* (1995). doi:10.2307/2346101

63. Li, Z. *et al.* Diverse and divergent protein post-translational modifications in two growth stages of a natural microbial community. *Nat Commun* **5,** 1–11 (2014).

64. Wiśniewski, J. R., Zougman, A., Nagaraj, N. & Mann, M. Universal sample preparation method for proteome analysis. *Nat. Methods* **6,** 359–362 (2009).

65. Washburn, M. P., Wolters, D. & Yates, J. R. Large-scale analysis of the yeast proteome by multidimensional protein identification technology. *Nature Biotechnology* **19,** 242–247 (2001).

66. Guo, X. *et al.* Sipros Ensemble improves database searching and filtering for complex metaproteomics. *Bioinformatics* **34,** 795–802 (2018).

67. Elias, J. E. & Gygi, S. P. Target-decoy search strategy for increased confidence in large-scale protein identifications by mass spectrometry. *Nat. Methods* **4,** 207–214 (2007).

68. Nesvizhskii, A. I. & Aebersold, R. Interpretation of shotgun proteomic data - The protein inference problem. *Molecular & Cellular Proteomics* **4,** 1419–1440 (2005).

69. Zhang, H. *et al.* dbCAN2: a meta server for automated carbohydrate-active enzyme annotation. *Nucleic Acids Res.* **46,** W95–W101 (2018).

70. Wu, Y.-W., Simmons, B. A. & Singer, S. W. MaxBin 2.0: an automated binning algorithm to recover genomes from multiple metagenomic datasets. *Bioinformatics* **32,** 605–607 (2016).

71. Alneberg, J. *et al.* Binning metagenomic contigs by coverage and composition. *Nat. Methods* **11,** 1144–1146 (2014).

72. Kang, D. D., Froula, J., Egan, R. & Wang, Z. MetaBAT, an efficient tool for accurately

reconstructing single genomes from complex microbial communities. *PeerJ* **3,** e1165–15 (2015).

73. Sieber, C. M. K. *et al.* Recovery of genomes from metagenomes via a dereplication, aggregation and scoring strategy. *Nature Microbiology* 1–10 (2018). doi:10.1038/s41564-018-0171-1

74. Parks, D. H., Imelfort, M., Skennerton, C. T., Hugenholtz, P. & Tyson, G. W. CheckM: assessing the quality of microbial genomes recovered from isolates, single cells, and metagenomes. *Genome Research* **25,** 1043–1055 (2015).

75. Parks, D. H. *et al.* Recovery of nearly 8,000 metagenome-assembled genomes substantially expands the tree of life. *Nature Microbiology* 1–10 (2017). doi:10.1038/s41564-017-0012-7

76. Stamatakis, A. RAxML version 8: a tool for phylogenetic analysis and post-analysis of large phylogenies. *Bioinformatics* **30,** 1312–1313 (2014).

77. Pruesse, E. *et al.* SILVA: a comprehensive online resource for quality checked and aligned ribosomal RNA sequence data compatible with ARB. *Nucleic Acids Res.* **35,** 7188–7196 (2007).

78. Taubert, M. *et al.* XoxF encoding an alternative methanol dehydrogenase is widespread in coastal marine environments. *Environmental Microbiology* **17,** 3937–3948 (2015).

79. Helen, D., Kim, H., Tytgat, B. & Anne, W. Highly diverse nirK genes comprise two major clades that harbour ammonium- producing denitrifiers. *BMC Genomics* 1–13 (2016). doi:10.1186/s12864-016-2465-0

80. Kruskal, W. H. & Wallis, W. A. Use of Ranks in One-Criterion Variance Analysis. *Journal of the American Statistical Association* **47,** 583–621 (1952).

81. WILCOXON, F. Individual Comparisons by Ranking Methods. *Biometrics Bulletin* **1,** 80–83 (1945).

82. R Core Team. *R: A Language and Environment for Statistical Computing*. 1–3604 (2014).

83. Society, R. F. J. O. T. R. S.1922. On the interpretation of χ 2 from contingency tables, and the calculation of P. *Journal of the Royal Statistical Society Series B …* **85,** 87 (1922).

84. Kursa, M. B. & Rudnicki, W. R. Feature Selection with the Boruta Package. *J. Stat. Soft.* **36,** 1–13 (2010).

85. WARD, J. H. Hierarchical Grouping to Optimize an Objective Function. *Journal of the American Statistical Association* **58,** 236–& (1963).
