# Supplementary material for: Mediterranean grassland soil C–N compound turnover is dependent on rainfall and depth, and is mediated by genomically divergent microorganisms
Source: Nat Microbiol. 2019 May 20;4(8):1356–67. doi: 10.1038/s41564-019-0449-y (PMC6784897; doi:10.1038/s41564-019-0449-y)
Supplement: Supplementary file 2 — Reporting Summary [file 41564_2019_449_MOESM2_ESM.pdf]

nature research

Corresponding author(s): Jillian F Banfield

Reporting Summary

Nature Research wishes to improve the reproducibility of the work that we publish. This form provides structure for consistency and transparency in reporting. For further information on Nature Research policies, see Authors & Referees and the Editorial Policy Checklist.

Statistical parameters

When statistical analyses are reported, confirm that the following items are present in the relevant location (e.g. figure legend, table legend, main text, or Methods section).

n/a	Confirmed	
☐	☒	The exact sample size (n) for each experimental group/condition, given as a discrete number and unit of measurement
☐	☒	An indication of whether measurements were taken from distinct samples or whether the same sample was measured repeatedly
☐	☒	The statistical test(s) used AND whether they are one- or two-sided<br>*Only common tests should be described solely by name; describe more complex techniques in the Methods section.*
☐	☒	A description of all covariates tested
☐	☒	A description of any assumptions or corrections, such as tests of normality and adjustment for multiple comparisons
☐	☒	A full description of the statistics including central tendency (e.g. means) or other basic estimates (e.g. regression coefficient) AND variation (e.g. standard deviation) or associated estimates of uncertainty (e.g. confidence intervals)
☐	☒	For null hypothesis testing, the test statistic (e.g. F, t, r) with confidence intervals, effect sizes, degrees of freedom and P value noted<br>*Give P values as exact values whenever suitable.*
☒	☐	For Bayesian analysis, information on the choice of priors and Markov chain Monte Carlo settings
☐	☒	For hierarchical and complex designs, identification of the appropriate level for tests and full reporting of outcomes
☐	☒	Estimates of effect sizes (e.g. Cohen's d, Pearson's r), indicating how they were calculated
☐	☒	Clearly defined error bars<br>*State explicitly what error bars represent (e.g. SD, SE, CI)*

Our web collection on statistics for biologists may be useful.

Software and code

Policy information about availability of computer code

Data collection	No software was used for data collection

Data analysis	FastQC v0.11.4<br>Sickle v1.33<br>IDBA_UD v1.1.0<br>Megahit v1.1.3<br>Prodigal v2.6.3<br>USEARCH v9.0<br>Bowtie2 v2.2.6<br>CONCOCT v0.4<br>MetaBAT v2<br>DAS Tool v1.1<br>CheckM v1.0.10<br>MUSCLE v3.8.31<br>MAFFT v7.310<br>FastTree v2.1<br>R v3.4.0. Specific R packages and statistical functions used in analysis are detailed in Methods.<br>Workflows describing the custom analysis code used in this study are available as described in the Code Availability Statement.

For manuscripts utilizing custom algorithms or software that are central to the research but not yet described in published literature, software must be made available to editors/reviewers upon request. We strongly encourage code deposition in a community repository (e.g. GitHub). See the Nature Research guidelines for submitting code & software for further information.

Data

Policy information about availability of data

All manuscripts must include a data availability statement. This statement should provide the following information, where applicable:

- Accession codes, unique identifiers, or web links for publicly available datasets
- A list of figures that have associated raw data
- A description of any restrictions on data availability

Genomic data including assemblies and raw reads will be made available under the NCBI BioProject accession number PRJNA449266.

Proteomic data are available through the ProteomeXchange Consortium via the PRIDE partner repository with identifier PXD013110.

Code involved in analysis will be made available at the following GitHub link: https://github.com/SDmetagenomics/Angelo2019_Paper.

A compressed archive of all genomes reconstructed in this study (See Supplementary Table 5) is also available here: https://www.dropbox.com/s/5iefsrtsi9ko2kr/Genomes.zip?dl=0

A compressed archive of all predicted proteins for genomes reconstructed in this study (See Supplementary Table 5) is also available here: https://www.dropbox.com/s/p4fb3ua0y0v21jd/Proteomes.zip?dl=0

Field-specific reporting

Please select the best fit for your research. If you are not sure, read the appropriate sections before making your selection.

☒ Life sciences ☐ Behavioural & social sciences ☐ Ecological, evolutionary & environmental sciences

For a reference copy of the document with all sections, see nature.com/authors/policies/ReportingSummary-flat.pdf

Life sciences study design

All studies must disclose on these points even when the disclosure is negative.

Sample size	The total sample size used for the metagenomics experiment was 60 samples, with a structured design of 3 sampling depths, 3 replicate plot locations, and 2 different treatment conditions, across 5 time points. The total sample size used for the proteomics experiments was 20 samples. Due to lower throughput of proteomics instrumentation and downstream analysis, samples were only collected for 2 sampling depths, 2 replicate plot locations, and 2 treatment conditions, across 3 time points. A formal analysis of statistical power was not performed, but these sample size were chosen based on an evaluation of sample sizes for microbial genome resolved metagenomics and proteomics experiments in existing literature, and made significantly larger to compare signals across different sample groupings.
Data exclusions	Two types of data were excluded from our analysis:<br>1) Sequencing reads with low quality scores, as is commonly performed prior to assembly of short read data.<br>2) Genomes were excluded from our bulk analysis of metabolism and statistical analysis of metabolic traits if they did not meet established criteria for completeness (>70 %) and contamination (<10 %) as measured by the checkM software package. This was done to limit false negatives when assigning functional information to genomes, and to assure that the genomes being analyzed are of similar and high quality.
Replication	> Sample Replication<br>All groupings of samples considered for statistical comparisons of genome abundance between samples contained > 10 biological replicates:<br>1) Depth: 10-20 cm (n = 24), 20-30 cm (n = 16), 30-40 cm (n = 20)<br>2) Treatment 10-20cm: Treatment 10-20 cm (n = 12) v. Control 10-20 cm (n = 12)

3) Treatment 30-40cm: Treatment 30-40 cm (n = 10) v Control 30-40 cm (n = 10)

We did not repeat the sampling, assembly, and analysis with a different set of soil samples, nor did we split samples and run two separate analysis. This was due to the cost of performing the initial experiment with large numbers of replicates, and the desire to maintain a high number of replicate samples for our statistical analysis respectively.

> Replication in Sampling Location:
The plots used for sampling consisted of 3 biological replicate plot pairs (control and treated with extended rainfall). We feel this level of replication was successful in showing both differences between physical plot locations as well as fine differences between control and treated plots. We specifically observe that rainfall treatment based effects were observed reproducibly in the context of plot location (which has a much stronger effect on organism distribution than treatment overall).

> Replication of Analyses Where Permutation was Used:
In some of our statistical analyses we applied permutation based methods (i.e. MRPP and enrichment permutation tests). Prior to reporting a final data value we repeated these analyses up to 5 times using different starting random seeds for the random number generation, and did not find any results changed during these tests. However, we only report a single result as we wanted to provide the same starting seed for all permutation based analyses, and seeds from test analyses were chosen at random internally by the computer as to avoid any bias in manual starting seed entry, and therefore were not recoverable. Thus, we feel outside of biological replication of the entire experiment, the testing of permutation based analyses before reporting a final result was successful in confirming that results were not obtained simply due to outliers generated by the randomization procedures.

Randomization

> Soil Plot Definition:
Soil plots of 70 m^2 circular sampling locations were laid out in a grid across the north meadow of the Angelo coast range reserve, CA, and plots that would receive extended rainfall treatment were selected as every other plot in the field. The pattern in plot layout, and treatment layout, was evenly distributed across the field and not randomized. Randomization was not performed in defining plots as there was a desire to have balanced numbers of plots from representative locations across the entire field site.

> Physical Soil Plot Sampling:
In our study soil plots were sampled at three depths, from paired plots, in triplicate. The exact sampling location within each plot that was sampled was randomly chosen for each set of cores that could include up to 3 depth strata, and any locations previously sampled were excluded on return sampling visits on different dates due to the destructive nature of the sampling. The longitudinal sampling dates were not randomized as we wanted these dates to fall at specific times before and after natural rainfall events.

> Defining Differentially Abundant Species Groups:
Species Groups (SGs; rpS3 markers clustered at 99% amino acid identity) were determined to be differentially abundant across depths, plots, and treatments using DEseq to assess differences in the counts of reads mapping to these sequences from each of our samples (see Replication for sample numbers). Randomization was not applied to the analysis of these groups as this is not a typical procedure for the analysis of grouped read count data. However, when analyzing the effect of a single variable such as depth or treatment response, we did control for co-variates using the linear modeling structure of the DEseq experimental design (i.e. Response = plot_replciate + treatment + date + depth -> in this case if we wanted to asses the effect of depth, the date of sampling, treatment status of the plot, and plot pair replicate would be controlled for)

> Determining Influence of Metadata Variables:
The statistical significance and strength of influence for plot location, treatment, depth, and time of sampling on the distribution of SGs was assessed using the multi response permutation procedure (MRPP). In this procedure samples were randomly associated with different metadata variables to determine significance and strength of influence (10,000 permutations). MRPP was performed in the vegan package in R and uses R internal random number generation for sample permutation. A seed was set in the code so that data is reproducible.

> Determining Phylum and Functional Enrichment Between Sample Groups:
The statistical significance of an observed distribution of a phylum or metabolic function was determined using a custom permutation function written in R, defined in the text, and available in the Github code (see Methods). For the group of genomes that made up a distribution during the testing (ie: all genomes that show differential abundance with depth), the observed distribution of these genomes with respect to a variable (ie: distribution of acidobacterial phylum genomes that increased or decreased with depth) was compared to randomly re-sampled sets, resulting the same number of genomes in permuted sets as the observed set, from the total set of genomes analyzed in our study (n = 793 genomes; 10,000 permutations per phylum or function test). Permutations were performed in R, and use R internal random number generation for sample permutation. A seed was set in the code so that data is reproducible.

> Randomization in Other Analyses:
In addition to the analyses explicitly listed, for other instances were permutation is mentioned in the text the randomization of samples was performed using R, explicitly the R internal random number generator. In all cases a seed was set to allow reproduction of results.

Blinding

Investigators were not blinded to group allocation during data analysis in this study. Initial Investigatory analysis of the data required the investigators to know the true groupings of the data to understand the results of data clustering and dimension reduction preformed at the onset of the analysis.

Reporting for specific materials, systems and methods

Materials & experimental systems

n/a	Involved in the study
☐	☒ Unique biological materials
☒	☐ Antibodies
☒	☐ Eukaryotic cell lines
☒	☐ Palaeontology
☒	☐ Animals and other organisms
☒	☐ Human research participants

Methods

n/a	Involved in the study
☒	☐ ChIP-seq
☒	☐ Flow cytometry
☒	☐ MRI-based neuroimaging

Unique biological materials

Policy information about availability of materials

Obtaining unique materials	Soil samples were collected from the south meadow field site at the Angelo Coast Range Reserve in northern California with permission given from APP# 27790; 39°44’21.4”N 123°37’51.0”W
